# Risk prediction model for precancerous gastric lesions based on magnifying endoscopy combined with narrow-band imaging features

**DOI:** 10.3389/fonc.2025.1554523

**Published:** 2025-04-04

**Authors:** Jingna Tao, Zhongmian Zhang, Linghan Meng, Liju Zhang, Jiaqi Wang, Zhihong Li

**Affiliations:** ^1^ Dongzhimen Hospital, Beijing University of Chinese Medicine, Beijing, China; ^2^ Beijing Hospital of Traditional Chinese Medicine, Capital Medical University, Beijing, China; ^3^ Guang’anmen Hospital, China Academy of Chinese Medical Sciences, Beijing, China

**Keywords:** precancerous gastric lesions, prediction model, Bayesian, random forest, XGBoost

## Abstract

**Background:**

This study aimed to construct and validate diagnostic models for the Operative Link on Gastritis Assessment (OLGA) and Operative Link on Gastric Intestinal Metaplasia Assessment (OLGIM) staging systems using three different methodologies based on magnifying endoscopy with narrow-band imaging (ME-NBI) features, to evaluate model performance, and to analyse risk factors for high-risk OLGA/OLGIM stages.

**Methods:**

We enrolled 356 patients who underwent white-light endoscopy and ME-NBI at the Department of Gastroenterology, Dongzhimen Hospital, Beijing University of Chinese Medicine, between January 2022 and September 2023. Clinical data were recorded. Chi-square or Fisher’s exact tests were used to analyse differences in endoscopic features between OLGA/OLGIM stages. Variables showing statistical significance underwent collinearity diagnosis before model inclusion. We constructed predictive models using Bayesian stepwise discrimination, random forest, and XGBoost algorithms. Receiver operating characteristic (ROC) curves were plotted using Python 3.12.4. Model accuracy, area under the ROC curve (AUC), sensitivity, and specificity were calculated for comprehensive validation.

**Results:**

All three models demonstrated excellent diagnostic performance, with random forest and XGBoost models showing marginally superior accuracy, AUC values, and sensitivity compared with the Bayesian stepwise discrimination model. For OLGA staging, the AUC values were 0.928, 0.958, and 0.966, with accuracies of 0.854, 0.902, and 0.918 for Bayesian, random forest, and XGBoost models, respectively. For OLGIM staging, the corresponding AUC values were 0.924, 0.975, and 0.979, with accuracies of 0.910, 0.938, and 0.927. Risk factors for high-risk OLGA included lesion location (subcardial and lower body greater curvature), intestinal metaplasia patches, lesion size, demarcation line (DL), and margin regularity of micro-capillary demarcation line (MCDL). Risk factors for high-risk OLGIM included *Helicobacter pylori* infection status, mucosal condition, lesion location (lesser curvature and lower body greater curvature), erosion, lesion size, DL, vessel and epithelial classification (VEC), white globe appearance (WGA), and MCDL margin regularity.

**Conclusions:**

All three models demonstrated robust accuracy and predictive capability, confirming that conventional white-light endoscopy combined with ME-NBI features provides valuable diagnostic reference for clinical risk assessment of precancerous gastric lesions.

## 1 Introduction

Gastric cancer is the fifth most common malignant tumour globally and ranks fourth among cancer-related deaths, accounting for 7.7% of total cancer mortality and posing a serious threat to human health (1). *Helicobacter pylori* (H. pylori) has been classified as a Group 1 carcinogen by the World Health Organisation and the International Agency for Research on Cancer consensus group (2). Studies have shown that *H. pylori* infection is associated with both intestinal and diffuse types of gastric cancer (3), making it the most common risk factor. *H. pylori* infection leads to persistent chronic active mucosal inflammation, potentially progressing to chronic atrophic gastritis (CAG), intestinal metaplasia (IM), and dysplasia (Dys) (4). Advanced stages of CAG, IM, and Dys are recognised as precancerous conditions (5, 6). Therefore, early and accurate diagnosis of precancerous gastric lesions is crucial for identifying high-risk patients.

The Operative Link on Gastritis Assessment (OLGA) and Operative Link on Gastric Intestinal Metaplasia Assessment (OLGIM) systems, evolved from the Sydney System (7, 8), reflect the severity of atrophy and intestinal metaplasia and assess gastric cancer risk. These systems have significant clinical value in gastric cancer screening and precancerous lesion surveillance (9). OLGA and OLGIM staging systems are based on histopathological examination following endoscopic forceps biopsy (EFB). Whilst histopathological examination remains the ‘gold standard’, this method presents notable limitations. EFB, being a point-sampling technique with limited specimen size, often fails to fully represent tumour cell heterogeneity, resulting in diagnostic deviation rates of 1.5%-8.0% (10–12). Although increasing the number of biopsies may enhance diagnostic accuracy, this approach can lead to mucosal injury and fibrosis, potentially compromising subsequent endoscopic interventions. Hence, there is an urgent need for a more comprehensive method to accurately determine lesion characteristics, extent, and identify high-risk mucosa. Endoscopic examination, compared to EFB histopathological assessment, offers the advantage of obtaining more comprehensive information about gastric mucosal lesions.

Magnifying endoscopy (ME) enables real-time observation of mucosal surface microstructures, whilst narrow-band imaging (NBI) is an emerging optical technology that aids in detecting early cancer and precancerous lesions (13, 14). The combination of ME and NBI provides clearer visualisation of fine mucosal surface microstructures and microvascular patterns, serving as a powerful tool for characterising gastric mucosal lesions and detecting early gastric cancer (15–17). The combination of conventional white-light endoscopy (C-WLE) and magnifying endoscopy with narrow-band imaging (ME-NBI) enables more sensitive detection of high-risk gastric mucosa through detailed mucosal characterisation. This approach also facilitates targeted EFB sampling, thereby improving the identification of precancerous gastric lesions. However, the predictive value of endoscopic mucosal features for high-risk OLGA/OLGIM stages is still uncertain, representing a critical gap in current diagnostic methods. This study is designed as both a development and validation study to address this gap by developing and validating diagnostic machine learning models. Indeed, the application of AI in the diagnosis and management of gastric diseases is rapidly expanding (18). To enhance OLGA/OLGIM risk stratification, artificial intelligence (AI) methodologies present a compelling advancement in diagnostic capabilities. Through systematic analysis of features obtained via magnifying endoscopy with narrow-band imaging (ME-NBI), AI systems can deliver more objective, consistent, and precise classification of precancerous lesion risk. This enhanced analytical approach facilitates more accurate patient risk stratification, potentially leading to optimised clinical decision-making and improved patient outcomes.

This study aims to stratify patients into OLGA/OLGIM stages and to construct and validate diagnostic models based on conventional white-light endoscopy (C-WLE) and ME-NBI features, employing Bayesian stepwise discrimination, random forest, and XGBoost algorithms. These models were selected for their complementary strengths: Bayesian models offer interpretability, random forests excel in feature selection, and XGBoost is renowned for its high predictive accuracy and ability to handle complex non-linear relationships, offering a comprehensive approach compared to relying solely on traditional endoscopic assessment or more complex ‘black-box’ deep learning models which may be less transparent and require larger datasets. Analysing endoscopic risk factors for high-risk OLGA/OLGIM patients will provide evidence for improved endoscopic identification of high-risk *H. pylori*-related precancerous lesions. The ultimate goal is to provide gastroenterologists with a practical, AI-enhanced tool that can improve the real-world clinical diagnosis of precancerous gastric lesions, potentially leading to more targeted biopsies, earlier detection of high-risk patients, and improved patient management strategies.

## 2 Materials and methods

### 2.1 Study population

This study collected clinical data from 356 patients who underwent C-WLE and ME-NBI examinations with histopathological results at the Endoscopy Centre of Dongzhimen Hospital, Beijing University of Chinese Medicine, between January 2022 and September 2023. Patient identifiers, including names, addresses, and contact information, were anonymised. The study was approved by the Ethics Committee of Dongzhimen Hospital, Beijing University of Chinese Medicine. The sample size (n=356) is comparable to similar machine learning studies in this field (19, 20). Whilst modest for complex models, especially with class imbalance, we employed SMOTE to mitigate this limitation. Larger, multi-centre validation is planned for future research.

### 2.2 Inclusion criteria

(1) Age ≥18 years
(2) Completion of both C-WLE and ME-NBI examinations
(3) Availability of standardised biopsy histopathological results suitable for OLGA/OLGIM staging
(4) Complete clinical data, including basic demographic characteristics, endoscopic findings, and pathological diagnoses

### 2.3 Exclusion criteria

(1) Autoimmune gastritis (type A chronic atrophic gastritis)
(2) Inadequate pathological sampling preventing OLGA/OLGIM staging
(3) Concurrent gastroduodenal ulcer or upper gastrointestinal bleeding
(4) Previous gastric surgery or history of any gastrointestinal tumours
(5) Other conditions deemed unsuitable for inclusion by investigators

These exclusion criteria were necessary to ensure a homogeneous study population focused on *H. pylori*-related precancerous lesions and to obtain reliable endoscopic and histopathological data. Specifically, autoimmune gastritis was excluded due to its distinct pathogenesis, whilst conditions such as ulcers and bleeding were excluded to prevent compromise of endoscopic image quality and biopsy accuracy. Whilst these exclusions strengthen internal validity, we acknowledge they may limit the model’s generalisability, which will be discussed further.

### 2.4 Data collection and quality control

**Figure 1:**
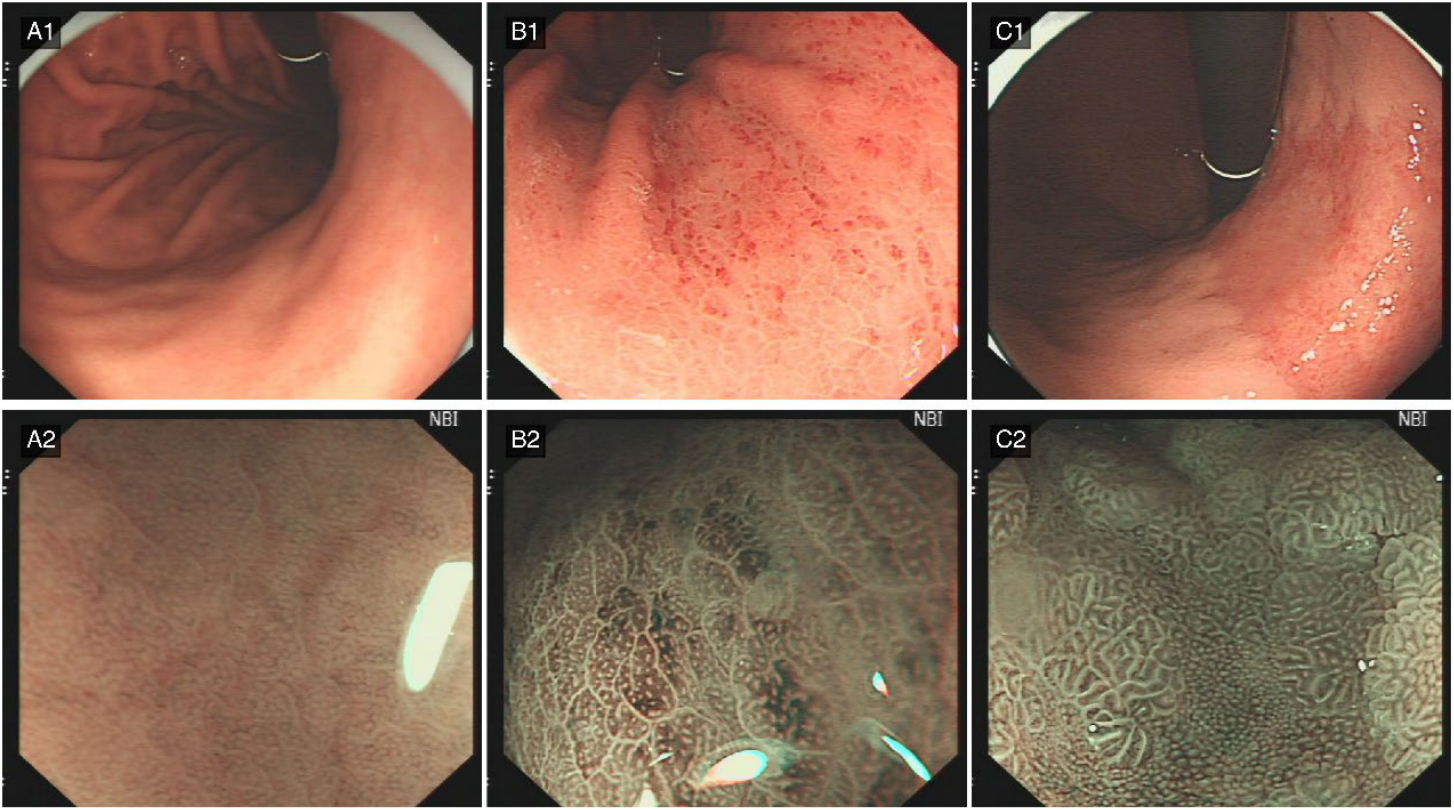
Characteristic features of *H*. *pylori* infection status. **(A)** Absence of Helicobacter pylori infection. **(A1)** (C-WLE): The gastric body mucosa exhibits a distinct regular arrangement of collecting venules (RAC), characteristically indicative of *H*. *pylori*-negative status. **(A2)** (ME-NBI): The gastric fundus glands display regular architectural arrangement with readily discernible glandular apertures. **(B)** Active Helicobacter pylori infection. **(B1)** (C-WLE): Diffuse mucosal redness manifests as extensive erythematous alterations throughout the gastric mucosa, suggesting active inflammatory processes. **(B2)** (ME-NBI): The inflammatory response is characterised by obscured glandular apertures accompanied by epithelial oedema at the glandular margins. **(C)** Post-Helicobacter pylori infection status. **(C1)** (C-WLE): Map-like redness presents as irregular erythematous regions within the gastric mucosa, displaying geographical patterning characteristic of post-eradication status. **(C2)** (ME-NBI): The mucosa demonstrates absence of normal glandular architecture concurrent with extensive intestinal metaplasia, corresponding to the observed map-like redness pattern.

**Figure 2:**
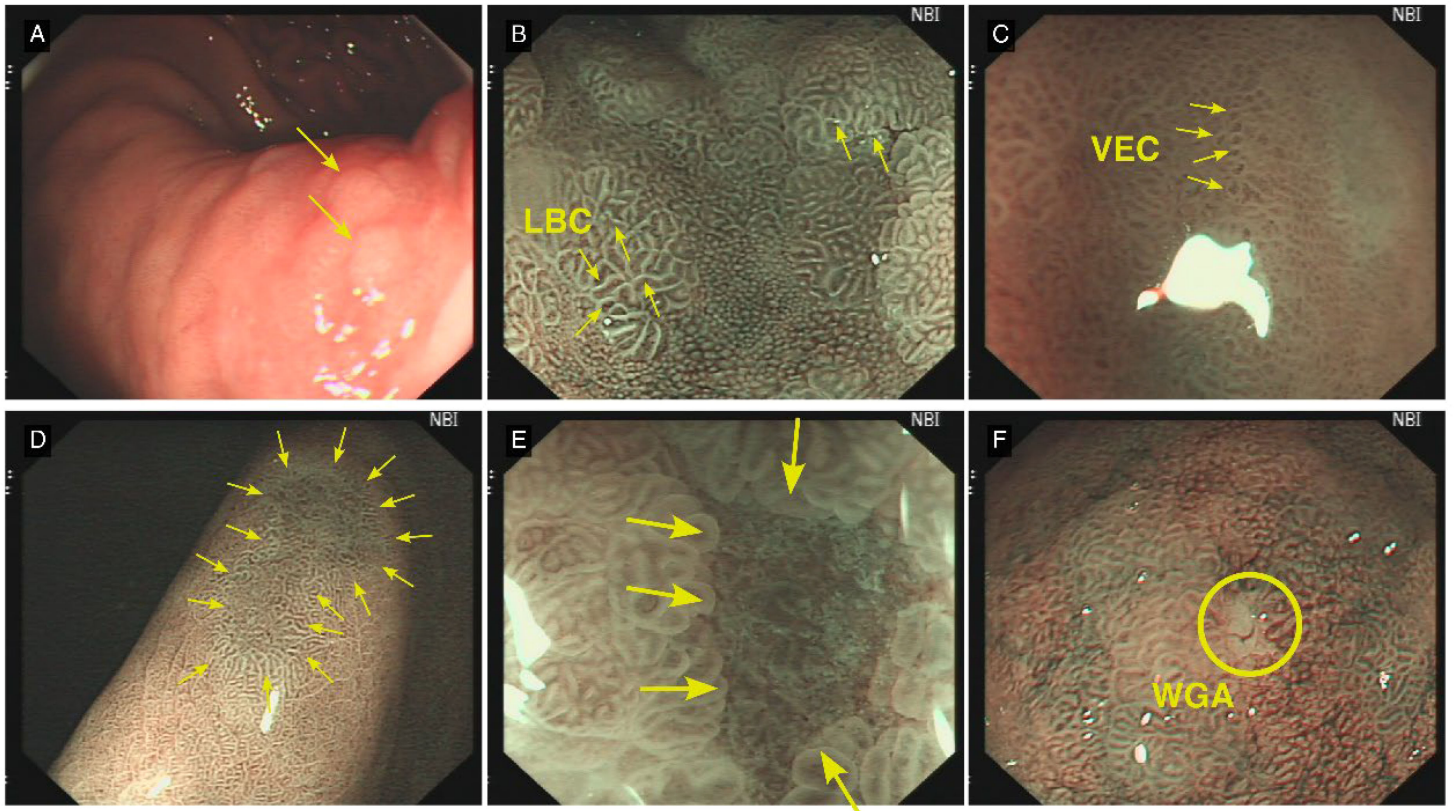
Typical Lesion Features Under C-WLE and ME-NBI. **(A)** (C-WLE): Intestinal metaplasia presents as a flat or marginally elevated white plaque (yellow arrow). **(B)** (ME-NBI): Light blue crest (LBC), a pathognomonic feature of intestinal metaplasia, manifests as delicate, light-blue linear structures or networks within the intervillous regions (yellow arrow). **(C)** (ME-NBI): The vessels within epithelial circle (VEC) pattern is observed, suggestive of potential dysplastic changes or early-stage carcinoma (yellow arrow). **(D)** A distinct demarcation line (DL) delineates the interface between the lesional and adjacent normal mucosal tissue (yellow arrow). **(E)** (ME-NBI): Multiple convex demarcation line (MCDL) demonstrates elevated demarcation boundaries at the epithelial margin of the surrounding mucosa. The morphological characteristics of these boundaries serve as crucial parameters in assessing lesional malignancy potential (yellow arrow). **(F)** (ME-NBI): White globe appearance (WGA) manifests as diminutive spherical white structures situated beneath the gastric epithelium. Histopathologically, these correspond to distended glandular lumina containing fragmentary necrotic epithelial debris (yellow circle).

1. Demographic characteristics: sex, age
2. Endoscopic lesion characteristics: According to the Kyoto Classification of Gastritis (21), C-WLE and ME-NBI features were described as follows:
  - *H. pylori* infection status (none, current, or past infection), **Figure 1**
  - Lesion location and orientation (subcardial, lesser curvature of the gastric body, gastric fundus, gastric angle, gastric antrum, anterior and posterior walls of the upper gastric body, greater curvature of the lower gastric body, pre-pyloric region)
  - C-WLE features: mucosal status (none, map-like redness, chicken skin appearance, diffuse redness, mucosal oedema), number of lesions, size, border clarity, gross morphology (elevated, flat, or depressed), colour (compared with surrounding mucosa: same, pale, or red), surrounding mucosal conditions (erosion, surface nodularity, ulceration, or intestinal metaplasia patches)
  - ME-NBI features: presence or absence of demarcation line (DL), irregular microvascular pattern (IMVP), irregular microsurface pattern (IMSP), light blue crest (LBC), white opaque substance (WOS), white globe appearance (WGA), vessels within epithelial circle (VEC) pattern, and multiple convex demarcation line (MCDL), as well as the clarity and regularity of the DL boundary (**Figure 2**).
3. Histopathological Diagnosis: According to the 2019 European Guidelines on Management of Precancerous Conditions and Lesions in the Stomach (22) and the 2022 Chinese Guidelines for Chronic Gastritis (23), histological results were staged with OLGA/OLGIM stages 0-II defined as low-risk and stages III-IV as high-risk.

To establish the reliability of endoscopic feature interpretation and histopathological staging, we conducted a comprehensive inter-observer agreement analysis. Two experienced endoscopists independently evaluated all images whilst blinded to clinical and pathological data. Concurrently, two senior pathologists independently performed OLGA/OLGIM staging. Any discrepancies were resolved through consensus discussion. Cohen’s kappa (κ) coefficients were calculated for all endoscopic features and staging classifications. The resultant analyses, detailed in the Results section, demonstrated exceptional agreement, thereby substantiating the robustness of both image interpretation and staging methodologies.

### 2.5 Research methods

(1) Feature Selection: Chi-square tests or Fisher’s exact tests were used to analyse correlations between endoscopic features and predictive variables (OLGA and OLGIM staging). Features with statistical significance (*P* < 0.05) were included in the candidate variable set for model construction.

(2) Collinearity Diagnosis: Following feature selection, variance inflation factor (VIF) analysis was performed to reduce the impact of highly collinear variables. A VIF threshold of 10 was used to identify potentially problematic collinearity, as this is a widely accepted criterion in many fields, including medical research (24, 25).

Variables with VIF > 10 were considered for removal or combination to ensure independence or low correlation among model variables. Specifically, VIF analysis identified demarcation line (DL), multiple convex demarcation line (MCDL) border size, and MCDL border regularity as having VIF values exceeding 10. However, given the clinical dependency of MCDL assessment on the presence of DL, and to maintain the clinical relevance of these features, all features, including those with high VIF values, were retained for model construction.

(3) Model Construction and Performance Evaluation: Three models were constructed: Bayesian stepwise discrimination, random forest, and XGBoost.

Initially, Bayesian stepwise discrimination analysis was performed using SPSS 30.0, employing stepwise variable introduction to generate discriminant functions. The model was validated through self-validation, leave-one-out cross-validation, and receiver operating characteristic (ROC) curve analysis.

Subsequently, random forest and XGBoost models were constructed using Python 3.12.4. Following data standardisation, Synthetic Minority Over-sampling Technique (SMOTE) was applied to address class imbalance. To assess the models’ generalisation capability, we implemented a systematic data partitioning protocol. The dataset underwent random stratification into a training cohort (comprising 80% of patients) and a testing cohort (comprising the remaining 20%). Stratified random sampling methodology was employed to preserve the proportional distribution of low-risk and high-risk OLGA/OLGIM cases across both cohorts. The training cohort facilitated model development, incorporating hyperparameter optimisation through 5-fold cross-validation protocols. The testing cohort was reserved exclusively for independent evaluation of the finalised models’ performance metrics. For robust validation, a 5-fold cross-validation was incorporated during the grid search process for hyperparameter tuning of both random forest and XGBoost models. Hyperparameter tuning for random forest and XGBoost models was conducted using grid search. For the random forest model, the tuned hyperparameters were ‘max_depth’: None, ‘min_samples_leaf’: 1, ‘min_samples_split’: 10, and ‘n_estimators’: 100. For the XGBoost model, the tuned hyperparameters were ‘colsample_bytree’: 0.8, ‘learning_rate’: 0.3, ‘max_depth’: 5, ‘n_estimators’: 100, and ‘subsample’: 0.8. Unless specified during grid search, default parameter settings in the scikit-learn and XGBoost libraries were used. Data were split into 80% training and 20% test sets. Grid search was employed for hyperparameter tuning of both models. The model validation protocol encompassed multiple metrics, including accuracy, sensitivity, specificity, AUC, F1-score and Precision-Recall curves. To address the inherent class imbalance, particular emphasis was placed on F1-scores and PR curves for evaluating performance in the minority high-risk OLGA/OLGIM classifications

External validation was not performed in this study due to its single-centre nature and the limitations in accessing external datasets. Future studies will focus on validating these models using multi-centre datasets to assess their generalizability. The dataset used in this study is available upon reasonable request from the corresponding author, subject to ethical approval and data protection regulations, as detailed in the ‘Availability of data and material’ section. The code for the developed machine learning models will be made available in a public repository upon publication to ensure reproducibility.

## 3 Results

### 3.1 Clinical demographic characteristics

The study included 356 patients with complete C-WLE, ME-NBI, and histopathological biopsy results. No significant differences were observed in sex and age distribution across OLGA/OLGIM stages (*P* > 0.05), as detailed in **Supplementary Table 1**.

**Supplementary Table 1** presents the demographic characteristics of the study cohort stratified by OLGA and OLGIM risk categories. No statistically significant differences in age or sex distribution were observed between low-risk and high-risk groups for either staging system (*P* > 0.05). This indicates demographic homogeneity across risk strata, suggesting these factors are unlikely to confound the analysis of endoscopic features.

The inter-observer agreement analysis revealed remarkable consistency between both endoscopists and pathologists. For OLGA-related assessments, Cohen’s kappa (κ) coefficients ranged from 0.849 (MCDL border regularity) to 1.000 (*H. pylori* infection status), with the majority of values exceeding 0.90. Analogously, OLGIM-related assessments yielded κ values ranging from 0.840 (prepyloric location) to 1.000 (*H. pylori* infection status), with most coefficients similarly surpassing 0.90. Notably, the κ values for OLGA and OLGIM staging demonstrated particularly high concordance at 0.953 and 0.948, respectively. These consistently elevated κ coefficients across all assessed parameters provide robust evidence for the reliability of both the endoscopic interpretation and histopathological staging protocols.

### 3.2 Comparison of C-WLE and ME-NBI features between OLGA stages

Among the 356 patients, 52 (14.6%) were classified as high-risk OLGA and 304 (85.4%) as low-risk. Under C-WLE, statistically significant differences between low-risk and high-risk OLGA patients were observed in *H. pylori* infection category, mucosal status, number of lesions, gross morphology, presence of intestinal metaplasia patches, and lesion size (all *P* < 0.001). Under ME-NBI, significant differences were found in the presence of DL (*P* < 0.001), presence of LBC (*P* = 0.003) and WOS (*P* = 0.002), MCDL border regularity (*P* < 0.001), and border size (*P* < 0.001) between OLGA stages (**Supplementary Tables 2**, **3**).

**Supplementary Table 2** compares C-WLE lesion characteristics between OLGA stages. Significant differences (*P* < 0.001) were found in *H. pylori* infection history, mucosal status, lesion number and size, morphology, IM patches. High-risk OLGA was associated with past *H. pylori*, map-like redness, multiple lesions, depressed morphology, IM patches, and larger size, highlighting macroscopically visible features indicative of advanced stages.

**Supplementary Table 3** details ME-NBI features and OLGA stage. Significant differences (*P* < 0.001) were observed for DL, MCDL border regularity and size, and LBC and WOS presence (*P* ≤ 0.003). High-risk OLGA showed increased DL, LBC, WOS, irregular MCDL border, and larger MCDL size, demonstrating ME-NBI’s ability to detect microstructural features associated with advanced OLGA.

### 3.3 Comparison of C-WLE and ME-NBI features between OLGIM stages

Of the 356 patients, 116 (32.6%) were classified as high-risk OLGIM and 240 (67.4%) as low-risk. Under C-WLE, statistically significant differences were found in *H. pylori* infection category, mucosal status, number of lesions, gross morphology, lesion border clarity, presence of intestinal metaplasia patches, and lesion size (all *P* < 0.001), lesion colour (*P* = 0.012), presence of erosion (*P* = 0.002), and surface nodularity (*P* = 0.032). Under ME-NBI, significant differences were observed in the presence of DL, LBC, WOS, and WGA, MCDL border regularity and size (all *P* < 0.001), and presence of VEC pattern (*P* = 0.006) between OLGIM stages (**Supplementary Tables 4**, **5**).

**Supplementary Table 4** presents C-WLE lesion characteristics by OLGIM stage. Significant differences (*P* < 0.05) were found for *H. pylori* history, mucosal status, lesion number and size, morphology, border clarity, IM patches, colour, erosion, and surface nodularity. High-risk OLGIM correlated with similar C-WLE features as high-risk OLGA, reinforcing the macroscopic endoscopic markers for advanced precancerous lesions.

**Supplementary Table 5** details ME-NBI features and OLGIM stage. Significant differences (P < 0.001) were observed for DL, LBC, WOS, WGA, MCDL border regularity and size, and VEC pattern (P = 0.006). High-risk OLGIM exhibited increased DL, LBC, WOS, WGA, irregular MCDL border, larger MCDL size, and VEC pattern, further emphasizing ME-NBI’s role in identifying microvascular and mucosal changes in advanced OLGIM stages.

### 3.4 Feature selection and collinearity diagnosis for C-WLE and ME-NBI characteristics

**Figure 3:**
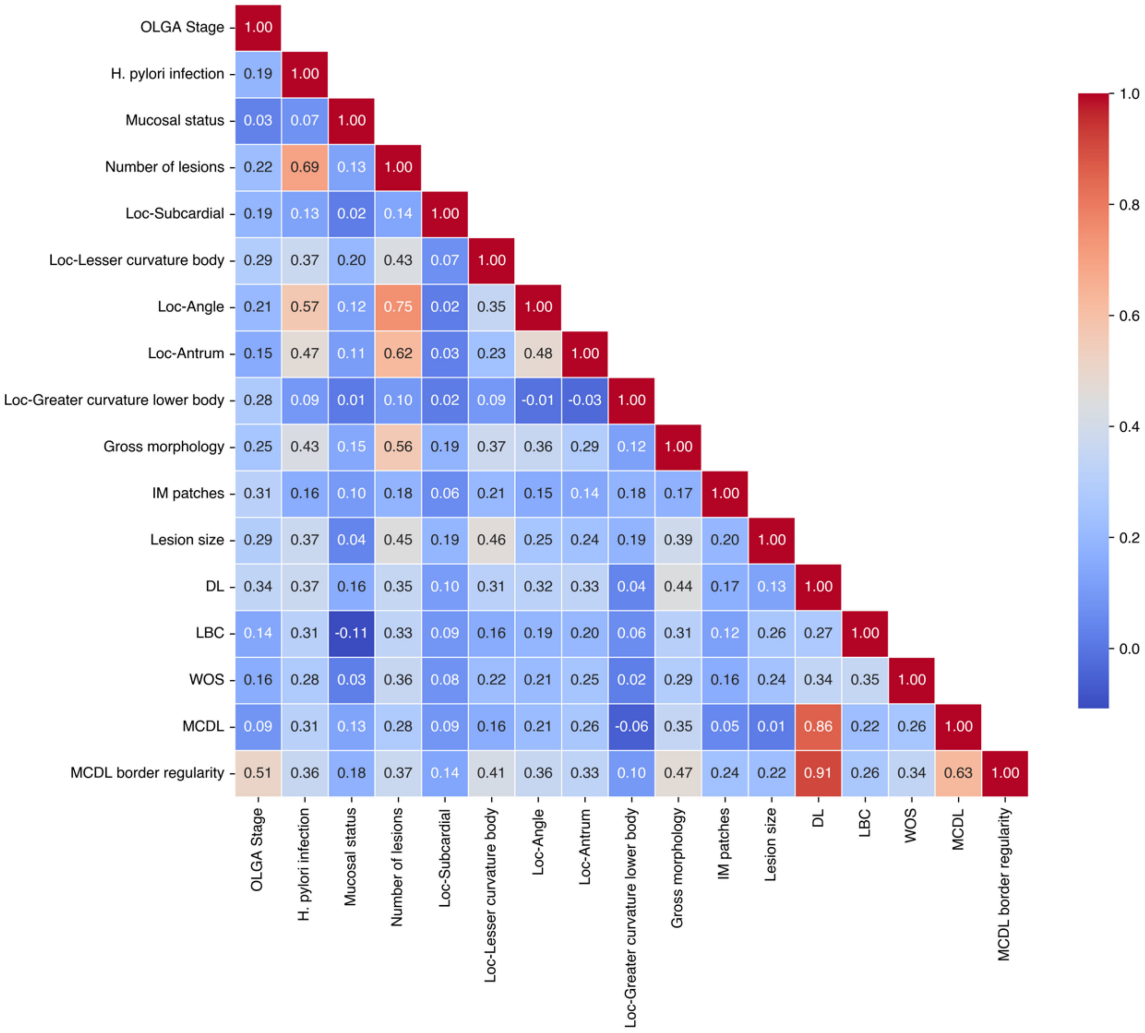
Correlation matrix analysis of endoscopic features based on OLGA staging.

**Figure 4:**
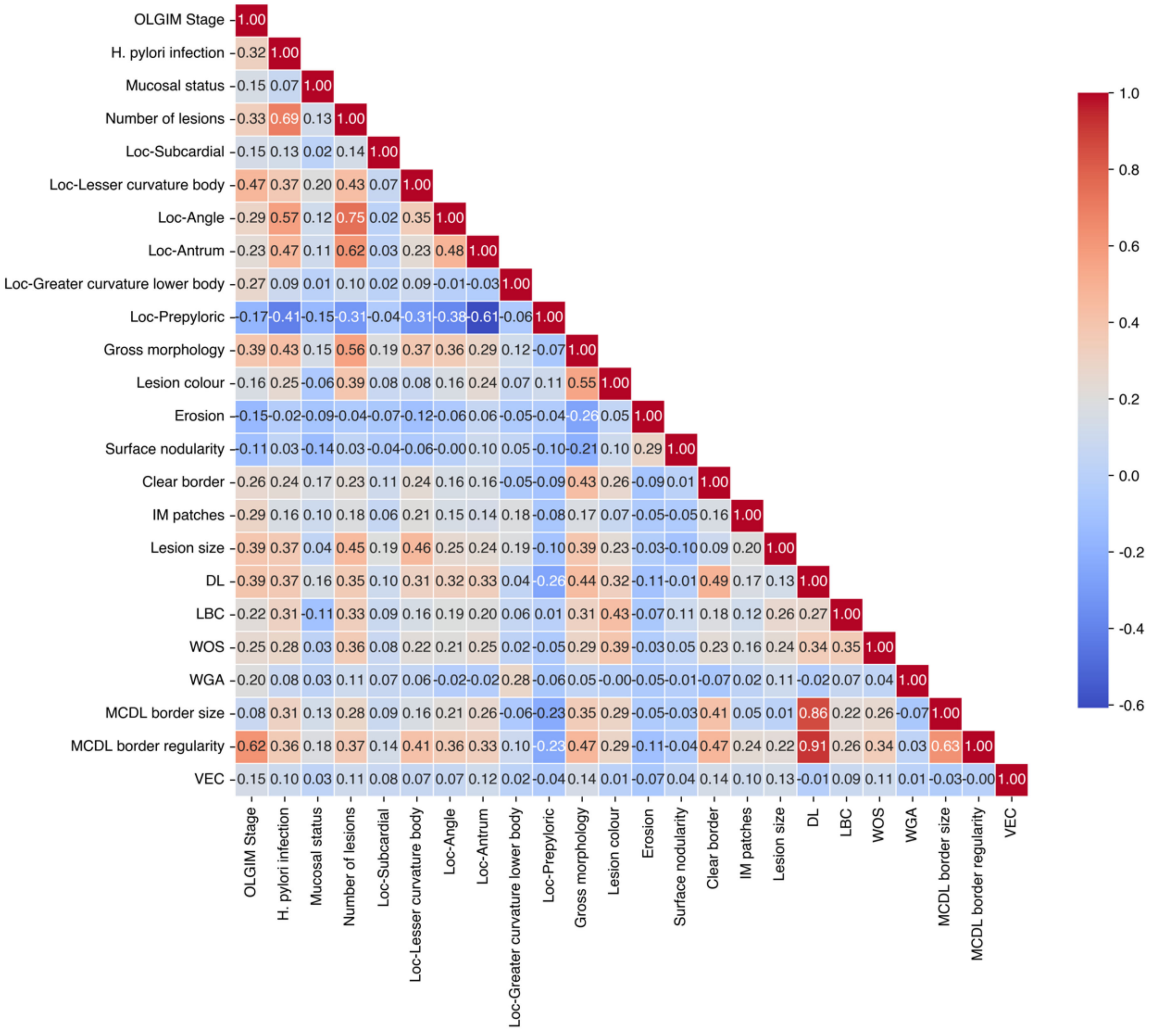
Correlation matrix analysis of endoscopic features based on OLGIM staging.

Based on chi-square and Fisher’s exact tests results, features significantly associated with OLGA/OLGIM staging from sections 2.2 and 2.3 were assigned values (**Table 1**). Collinearity analysis was performed on these features (**Tables 2**, **3**), with final variable selection requiring VIF < 10. DL, MCDL border size, and border regularity showed VIF > 10, confirmed by feature correlation matrix analysis (**Figures 3**, **4**). However, given that MCDL assessment clinically depends on DL presence, indicating a structural dependency, these features were retained for subsequent model construction.

**Table 1:** Endoscopic feature variable coding.

Endoscopic Feature	Coding
*H. pylori* infection (X1)	0=None, 1=Current, 2=Past
Mucosal status (X2)	0=None, 1=Map-like redness, 2=Patchy redness, 3=Chicken skin appearance, 4=Diffuse redness, 5=Mucosal oedema
Gross morphology (X3)	0=None, 1=Elevated, 2=Flat, 3=Depressed
Number of lesions (X4)	0=None, 1=Single, 2=Multiple
Lesion colour (X5)	0=None, 1=Same, 2=Pale, 3=Red
Location-Subcardial (X6)	0=Absent, 1=Present
Location- Lesser curvature of the gastric body (X7)	0=Absent, 1=Present
Location- Greater curvature of the lower gastric body (X8)	0=Absent, 1=Present
Location-Gastric angle (X9)	0=Absent, 1=Present
Location- Gastric antrum (X10)	0=Absent, 1=Present
Location-Pre-pyloric region (X11)	0=Absent, 1=Present
Clear border (X12)	0=Absent, 1=Present
Erosion (X13)	0=Absent, 1=Present
Surface nodularity (X14)	0=Absent, 1=Present
IM patches (X15)	0=Absent, 1=Present
Lesion size (X16)	0=None, 1=<1cm, 2=≥1cm
DL (X17)	0=Absent, 1=Present
LBC (X18)	0=Absent, 1=Present
WOS (X19)	0=Absent, 1=Present
VEC (X20)	0=Absent, 1=Present
WGA (X21)	0=Absent, 1=Present
MCDL border size (X22)	0 = 0, 1=>0 to <1/3, 2=≥1/3 to <2/3, 3=≥2/3
MCDL border regularity (X23)	0=None, 1=Regular, 2=Irregular

**Table 2:** Collinearity analysis of endoscopic features based on OLGA staging.

Model	Unstandardised Coefficients		Standardised Coefficients	t	P-value	Collinearity Statistics	
	B	Standard Error	β			Tolerance	VIF
(Constant)	0.015	0.066		0.222	0.824		
*H. pylori* infection	-0.009	0.030	-0.018	-0.296	0.768	0.475	2.106
Mucosal status	-0.023	0.014	-0.070	-1.616	0.107	0.900	1.111
Number of lesions	-0.025	0.061	-0.037	-0.407	0.684	0.207	4.835
Location-Subcardial	0.156	0.063	0.106	2.478	0.014	0.912	1.096
Location-Lesser curvature of the gastric body	-0.001	0.037	-0.001	-0.025	0.980	0.622	1.608
Location-Gastric angle	0.040	0.051	0.052	0.789	0.430	0.384	2.606
Location-Gastric antrum	0.007	0.050	0.008	0.144	0.885	0.565	1.769
Location-Greater curvature of the lower gastric body	0.329	0.085	0.168	3.860	0.000	0.885	1.129
Morphology	-0.013	0.025	-0.030	-0.538	0.591	0.547	1.829
IM patches	0.153	0.049	0.135	3.096	0.002	0.877	1.140
Lesion size	0.059	0.032	0.097	1.852	0.065	0.618	1.618
DL	-0.335	0.174	-0.474	-1.924	0.055	0.028	36.138
LBC	-0.012	0.054	-0.011	-0.230	0.819	0.761	1.314
WOS	-0.012	0.040	-0.015	-0.309	0.757	0.750	1.333
MCDL border size	-0.020	0.037	-0.070	-0.546	0.586	0.101	9.877

**Table 3:** Collinearity analysis of endoscopic features based on OLGIM staging.

Model	Unstandardised Coefficients		Standardised Coefficients	t	P-value	Collinearity Statistics	
	B	Standard Error	β			Tolerance	B
(Constant)	-0.069	0.072		-0.963	0.336		
*H. pylori* infection	0.063	0.032	0.094	1.985	0.048	0.437	2.286
Mucosal status	0.007	0.015	0.015	0.447	0.655	0.871	1.147
Number of lesions	-0.061	0.067	-0.068	-0.915	0.361	0.176	5.680
Location-Subcardial	0.015	0.065	0.008	0.234	0.815	0.895	1.117
Location-Lesser curvature of the gastric body	0.096	0.040	0.103	2.435	0.015	0.553	1.810
Location-Gastric angle	0.020	0.054	0.020	0.370	0.712	0.346	2.886
Location-Gastric antrum	0.023	0.066	0.019	0.353	0.725	0.338	2.962
Location-Greater curvature of the lower gastric body	0.308	0.091	0.119	3.372	0.001	0.795	1.258
Location-Pre-pyloric region	-0.024	0.081	-0.015	-0.297	0.767	0.411	2.433
Morphology	-0.016	0.032	-0.027	-0.497	0.619	0.345	2.897
Colour	0.024	0.030	0.037	0.798	0.425	0.460	2.175
Erosion	-0.105	0.069	-0.054	-1.531	0.127	0.788	1.269
Surface nodularity	-0.095	0.060	-0.058	-1.587	0.113	0.747	1.339
Clear border	0.034	0.040	0.034	0.860	0.390	0.647	1.545
IM patches	0.055	0.051	0.037	1.087	0.278	0.862	1.160
Lesion size	0.058	0.033	0.071	1.751	0.081	0.596	1.677
DL	-0.870	0.183	-0.928	-4.762	0.000	0.026	38.454
LBC	0.039	0.058	0.025	0.669	0.504	0.694	1.440
WOS	0.026	0.042	0.023	0.609	0.543	0.714	1.400
WGA	0.210	0.082	0.087	2.558	0.011	0.852	1.173
MCDL border size	-0.009	0.039	-0.022	-0.219	0.827	0.095	10.483
MCDL border regularity	0.743	0.073	1.337	10.193	0.000	0.057	17.445
VEC	0.196	0.064	0.101	3.040	0.003	0.903	1.108

**Table 1** outlines the coding scheme for endoscopic features used in subsequent statistical analyses and model construction. This standardisation ensures consistent variable representation for quantitative analysis and facilitates model interpretability.

**Table 2** presents the collinearity analysis for endoscopic features related to OLGA staging. High VIF values (>10) for DL indicate multicollinearity, while the VIF values for MCDL border size are close to 10 (9.877), suggesting a potential concern for multicollinearity. Despite this, these features were retained due to the clinical dependency of MCDL assessment on DL presence, reflecting their structural relationship in endoscopic evaluation.

**Table 3** shows the collinearity analysis for endoscopic features related to OLGIM staging. Similar to OLGA, high VIF values (>10) were observed for DL, MCDL border size, and border regularity, indicating multicollinearity. These features were retained due to their clinical relevance and structural dependency in endoscopic assessment, despite statistical collinearity.

### 3.5 Construction and validation of Bayesian stepwise discrimination model

To develop high-performance predictive models for precancerous gastric lesion risk, we employed rigorous feature selection criteria encompassing four key dimensions: statistical significance (*P* < 0.05 between low-risk and high-risk cohorts), clinical relevance (demonstrated association with OLGA/OLGIM staging pathophysiology or established diagnostic utility from previous investigations), endoscopic feasibility (reliable assessment during routine C-WLE and ME-NBI examinations), and multicollinearity considerations (generally VIF < 10).

The selected features represent complementary diagnostic modalities: C-WLE features, including mucosal status, lesion size/morphology, and IM patches, characterise macroscopic mucosal alterations, whilst ME-NBI features, comprising demarcation line, light blue crest, white opaque substance, MCDL border regularity, and VEC, elucidate microstructural details. This dual-modality approach yields a comprehensive representation of pathological alterations, thereby enhancing the model’s predictive capability for high-risk OLGA/OLGIM stages.

Features excluded from the final models met one or more elimination criteria: insufficient statistical significance (*P* ≥ 0.05), excessive multicollinearity (generally VIF > 10; notably, DL, MCDL border size, and regularity were retained owing to the clinical dependency of MCDL assessment on DL presence), inadequate clinical significance or assessment feasibility.

Based on features selected in section 3.4, comprehensive diagnostic models for OLGA/OLGIM staging of precancerous gastric lesions were established. Variables X1, X2, X3, X4, X6, X7, X8, X9, X10, X15, X16, X17, X18, X19, X22, and X23 were used for the OLGA staging diagnostic model, whilst X1 through X23 were used for the OLGIM staging model.

#### 3.5.1 Bayesian stepwise discrimination analysis model

Using stepwise forward selection in Bayesian stepwise discrimination analysis, classification function coefficients for OLGA and OLGIM staging models were obtained (**Tables 4**, **5**), yielding the following prediction model equations:

**Table 4:** Classification function coefficients for stepwise discriminant model of OLGA staging.

Feature Variable	Low-risk OLGA	High-risk OLGA
Location-Subcardial	0.626	2.789
Location-Greater curvature of the lower gastric body	0.655	5.346
IM patches	0.557	2.696
DL	2.163	-4.139
MCDL border regularity	-0.250	5.696
(Constant)	-1.101	-5.021

**Table 5:** Classification function coefficients for stepwise discriminant model of OLGIM staging.

Feature Variable	Low-risk OLGIM	High-risk OLGIM
*H. pylori* infection status	2.443	3.196
Location-Lesser curvature of the gastric body	-0.819	0.424
Location-Greater curvature of the lower gastric body	-1.120	2.898
Erosion	1.085	-0.621
Size	3.523	4.380
DL	1.615	-10.463
WGA	0.222	2.888
MCDL border regularity	-0.583	9.806
VEC	-0.284	2.485
(Constant)	-4.668	-11.422


$$\begin{array}{l} \text{OLGA low}-\text{risk}=-1.101+0.626\text{X}6+0.655\text{X}8\\ +0.557\text{X}15+2.163\text{X}16-0.25\text{X}23 \end{array}$$

$$\begin{array}{l} \text{OLGA high}-\text{risk}=-5.021+2.789\text{X}6+5.346\text{X}8\\ +2.696\text{X}15-4.139\text{X}16+5.696\text{X}23 \end{array}$$

$$\begin{array}{l} \text{OLGIM low}-\text{risk }\\ =\;-4.668\;+\;2.443X1\;-\;0.819\text{X}7\;-\;1.12\text{X}8\;+\;1.085\text{X}13\;\\ +\;3.523\text{X}16\;+\;1.615\text{X}17\;-\;0.284\text{X}20\;+\;0.222\text{X}21\;\\ -\;0.583\text{X}23 \end{array}$$

$$\begin{array}{l} \text{OLGIM high}-\text{risk }\\ =\;-11.442\;+\;3.196\text{X}1\;+\;0.424\text{X}7\;+\;2.898\text{X}8\;-\;0.621\text{X}13\;\\ +\;4.38\text{X}16\;-\;10.463\text{X}17\;+\;2.485\text{X}20\;+\;2.888\text{X}21\;\\ +\;9.806\text{X}23 \end{array}$$

**Table 4** displays the classification function coefficients derived from the Bayesian stepwise discriminant model for OLGA staging. These coefficients quantify the contribution of each selected endoscopic feature (location-subcardial, location-greater curvature of lower body, IM patches, DL, MCDL border regularity) to the prediction of low-risk and high-risk OLGA stages within the Bayesian model.

**Table 5** presents the classification function coefficients from the Bayesian stepwise discriminant model for OLGIM staging. These coefficients indicate the weight of each selected endoscopic feature (H. pylori status, location-lesser curvature and greater curvature of lower body, erosion, size, DL, WGA, MCDL border regularity, VEC) in predicting low-risk and high-risk OLGIM stages within the Bayesian model.

#### 3.5.2 Validation of Bayesian discrimination model

For self-validation, the overall accuracy rates were 85.4% for the OLGA staging prediction model and 91.0% for the OLGIM staging prediction model. In cross-validation, the overall accuracy rates were 85.1% for OLGA and 91.0% for OLGIM. Both models demonstrated high accuracy (**Table 6**).

**Table 6:** Bayesian stepwise discriminant results for OLGA/OLGIM staging diagnostic models (Self-validation and Cross-validation).

Validation Method	Predictive Model	Overall Accuracy	Low-risk Accuracy	High-risk Accuracy
Self-validation	OLGA	85.4%	83.9%	94.2%
	OLGIM	91.0%	96.3%	80.2%
Cross-validation	OLGA	85.1%	83.9%	92.3%
	OLGIM	91.0%	96.3%	80.2%

**Table 6** summarises the validation results of the Bayesian stepwise discriminant models for OLGA and OLGIM staging. Both self-validation and cross-validation demonstrated high overall accuracy (OLGA: ~85%, OLGIM: ~91%), indicating robust performance of the Bayesian models in classifying precancerous gastric lesions.

**Figure 5:**
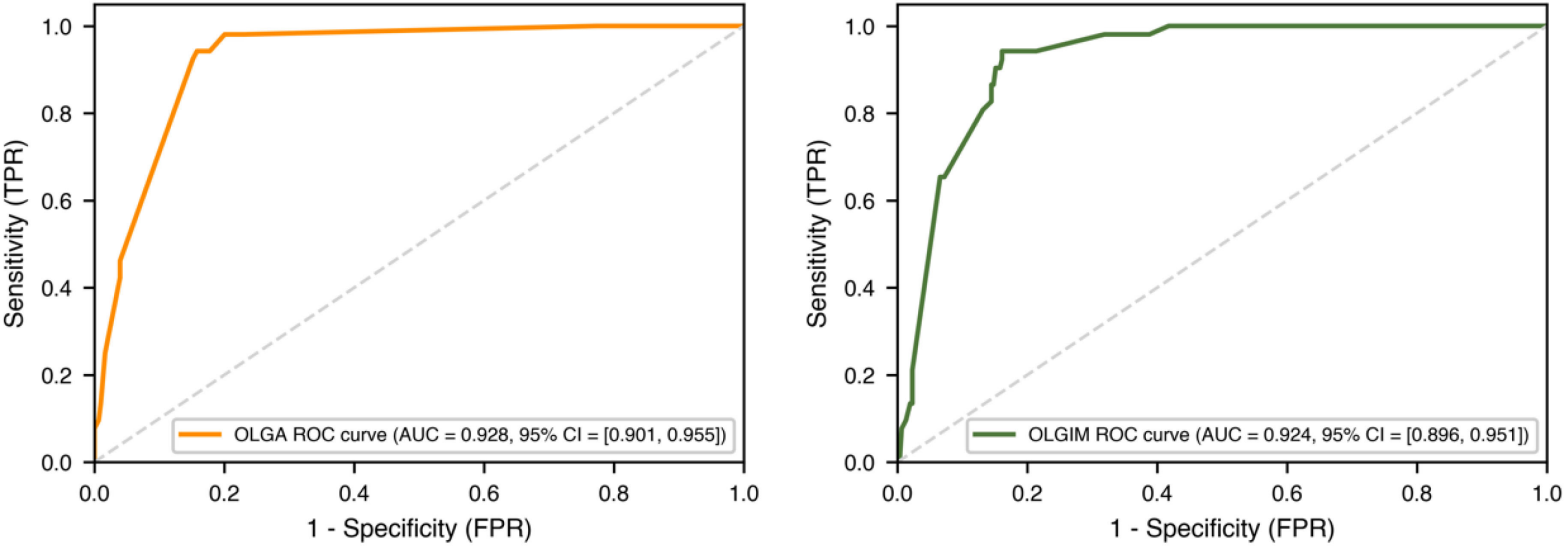
ROC curves for bayesian stepwise discriminant OLGA and OLGIM staging diagnostic models.

ROC curves were constructed to evaluate the performance of both prediction models (**Figure 5**), with detailed parameters shown in **Table 7**. The OLGA model achieved an AUC value of 0.928 (95% CI: 0.901-0.955), with sensitivity of 0.942 and specificity of 0.842. The OLGIM model achieved an AUC value of 0.924 (95% CI: 0.896-0.951), with sensitivity of 0.942 and specificity of 0.839.

**Table 7:** Performance of bayesian stepwise discriminant OLGA and OLGIM staging diagnostic models.

Model	AUC	95% CI	Sensitivity	Specificity
OLGA Model	0.928	0.901-0.955	0.942	0.842
OLGIM Model	0.924	0.896-0.951	0.942	0.839

**Table 7** details the performance metrics of the Bayesian models. Both OLGA and OLGIM models achieved high AUC values (~0.92), sensitivity (~0.94), and specificity (~0.84), confirming their excellent discriminatory ability for identifying high-risk precancerous gastric lesions based on Bayesian stepwise discrimination.

### 3.6 Construction and validation of random forest model

#### 3.6.1 Establishment of OLGA/OLGIM staging diagnostic models based on random forest

Random forest algorithms were employed to construct prediction models for both OLGA and OLGIM staging. Following data standardisation and class balancing, Synthetic Minority Over-sampling Technique (SMOTE) was applied to address the imbalance between low-risk and high-risk classifications. Grid search and cross-validation methods were utilised during model training to optimise parameters for optimal predictive performance.

#### 3.6.2 Random forest model performance

**Figure 6:**
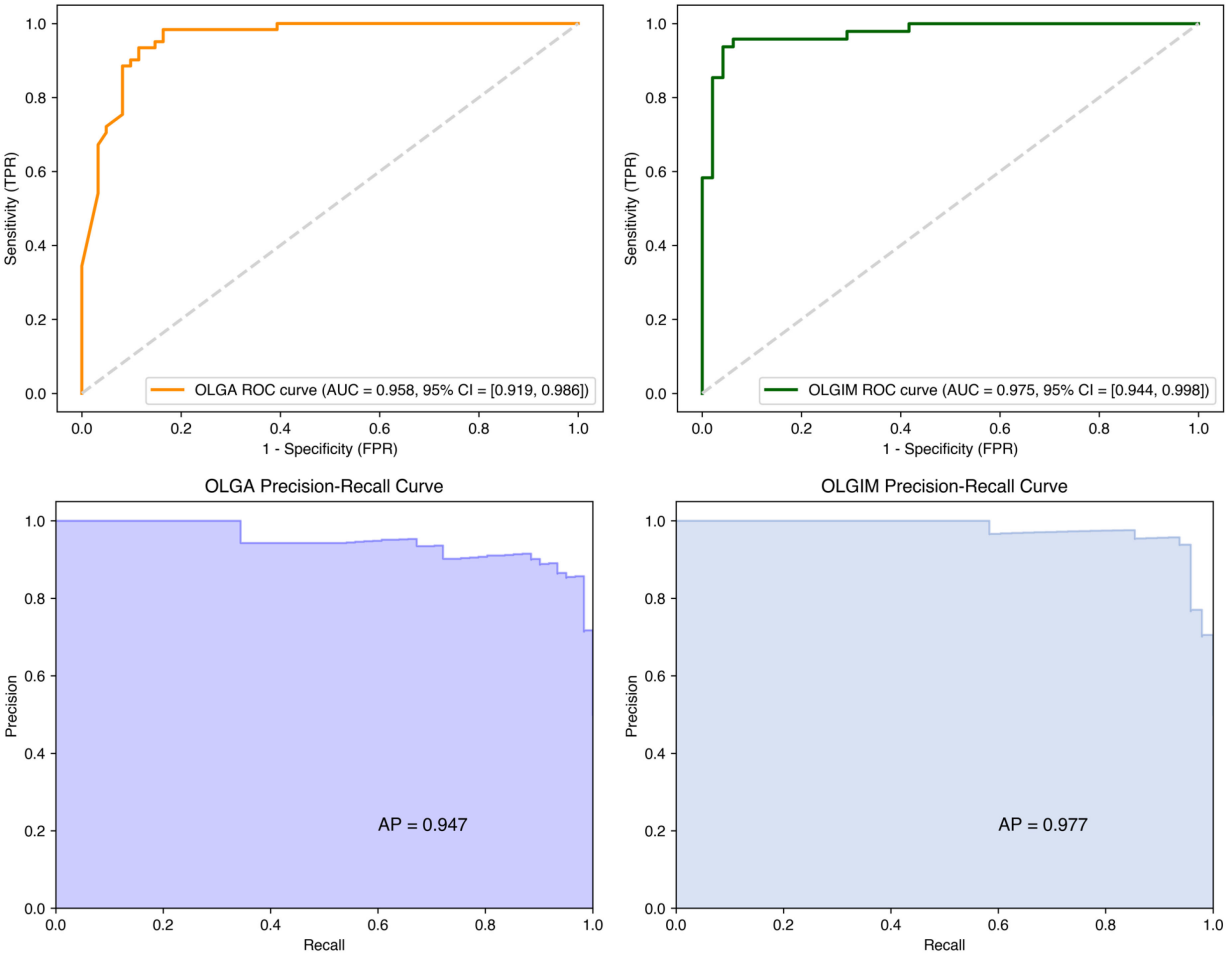
ROC and precision-recall curves for random forest OLGA and OLGIM staging diagnostic models.

ROC curves were constructed to evaluate model performance. On the test set, the OLGA and OLGIM staging diagnostic models achieved accuracy rates of 0.902 and 0.938, respectively; AUC values of 0.958 and 0.975; precision of 0.855 and 0.938; sensitivity of 0.967 and 0.938; specificity of 0.836 and 0.938; and F1 scores of 0.908 and 0.938 (**Table 8**). The ROC curves for both models deviated substantially from the diagonal line, indicating robust predictive performance (**Figure 6**).

**Table 8:** Performance of random forest OLGA and OLGIM staging diagnostic models.

Model	Accuracy	AUC	Specificity	Sensitivity	Precision	F1 Score
OLGA Model	0.902	0.958	0.836	0.967	0.855	0.908
OLGIM Model	0.938	0.975	0.938	0.938	0.938	0.938

**Table 8** presents the performance metrics of the Random Forest models. Both OLGA and OLGIM models demonstrated high accuracy (~0.90 and ~0.94 respectively) and AUC values (~0.96 and ~0.975 respectively), along with balanced sensitivity and specificity, indicating strong predictive capability for risk stratification using Random Forest algorithms.

The Random Forest models demonstrated robust performance in high-risk classification, achieving F1-scores of 0.908 (OLGA) and 0.938 (OLGIM). **Figure 6** presents the Precision-Recall curves, which elucidate the precision-recall trade-off for high-risk OLGA/OLGIM classifications.

#### 3.6.3 Random forest model stability assessment

**Figure 7:**
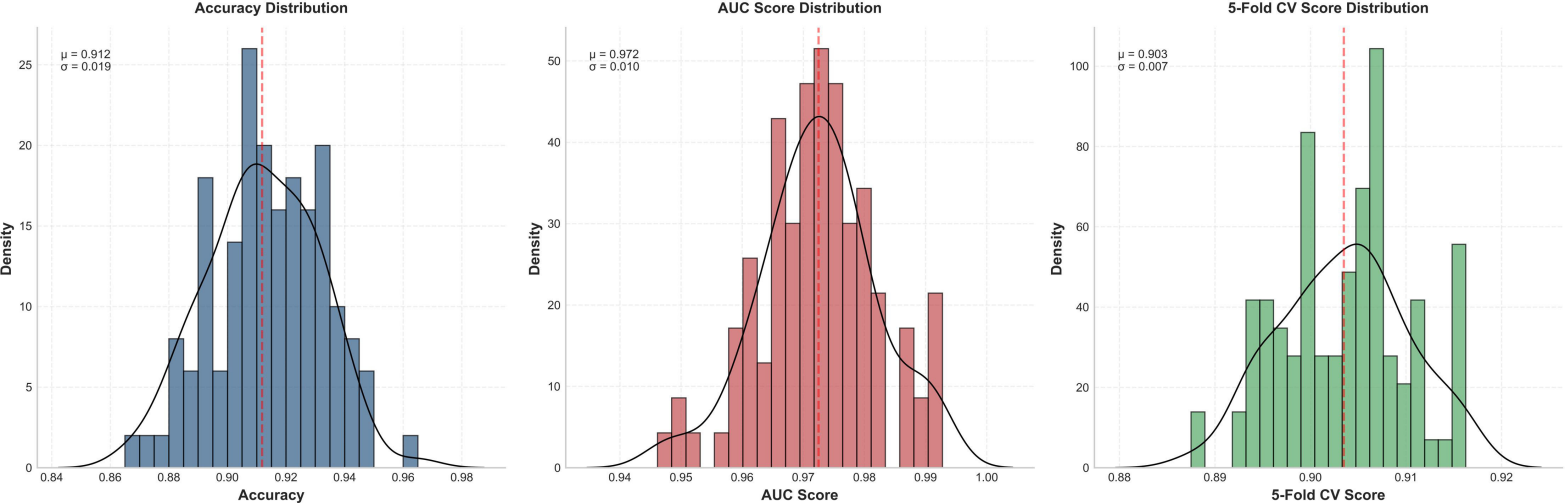
Stability assessment of the random forest OLGA staging diagnostic model.

**Figure 8:**
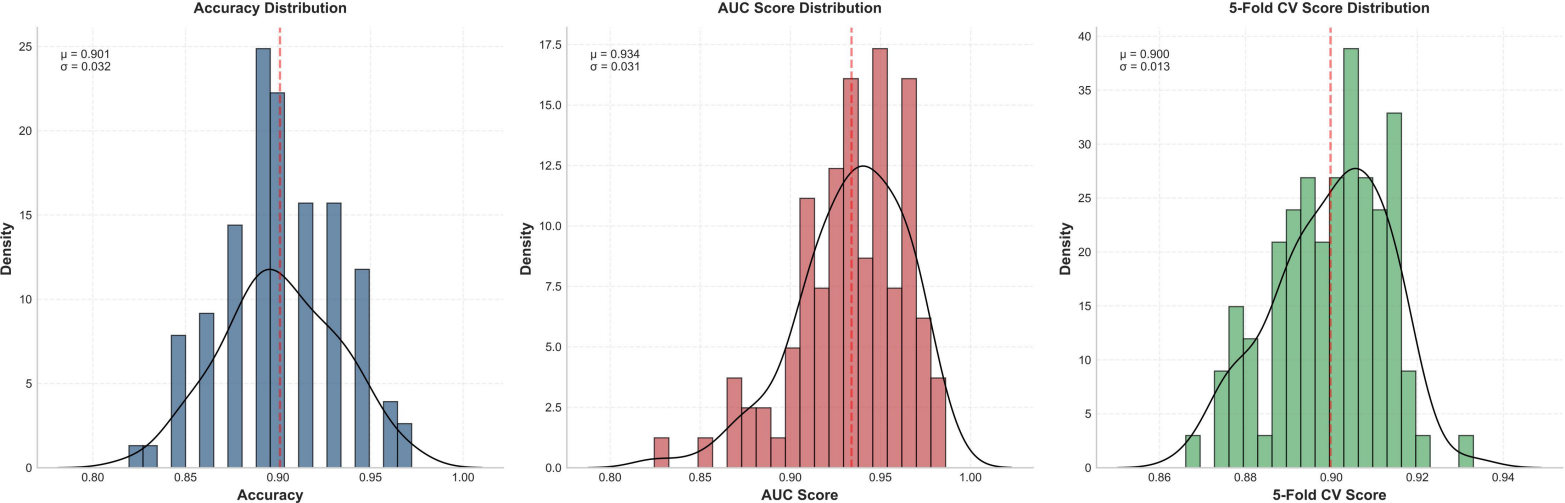
Stability assessment of the random forest OLGIM staging diagnostic model.

To comprehensively evaluate the reliability of predictive results, we conducted thorough stability testing. Through 100 random sampling evaluations and 5-fold cross-validation, we obtained statistical distributions of model performance metrics. The OLGA model achieved a mean accuracy of 0.912 ± 0.019 and mean AUC value of 0.972 ± 0.010, whilst the OLGIM model achieved a mean accuracy of 0.901 ± 0.032 and mean AUC value of 0.934 ± 0.031. The small standard deviations indicate stable predictive performance across different data subsets. Furthermore, 5-fold cross-validation revealed mean validation scores of 0.903 ± 0.007 for the OLGA model and 0.900 ± 0.013 for the OLGIM model. This stable cross-validation performance further confirms model reliability, demonstrating consistent high predictive accuracy across different patient populations (**Figures 7**, **8**).

#### 3.6.4 Analysis of key predictive features in random forest models

Feature importance analysis revealed the relative contributions of different endoscopic characteristics to the prediction models. In the OLGA model, the top three features were MCDL border regularity (28.15%), MCDL border size (15.64%), and presence of DL (14.42%). For the OLGIM model, the most important features were MCDL border regularity (25.84%), mucosal status (15.75%), and MCDL border size (10.77%). These results indicate that these specific endoscopic features have high predictive value for assessing precancerous gastric lesion risk (**Supplementary Figure 1**, **2**).

**Figure 9:**
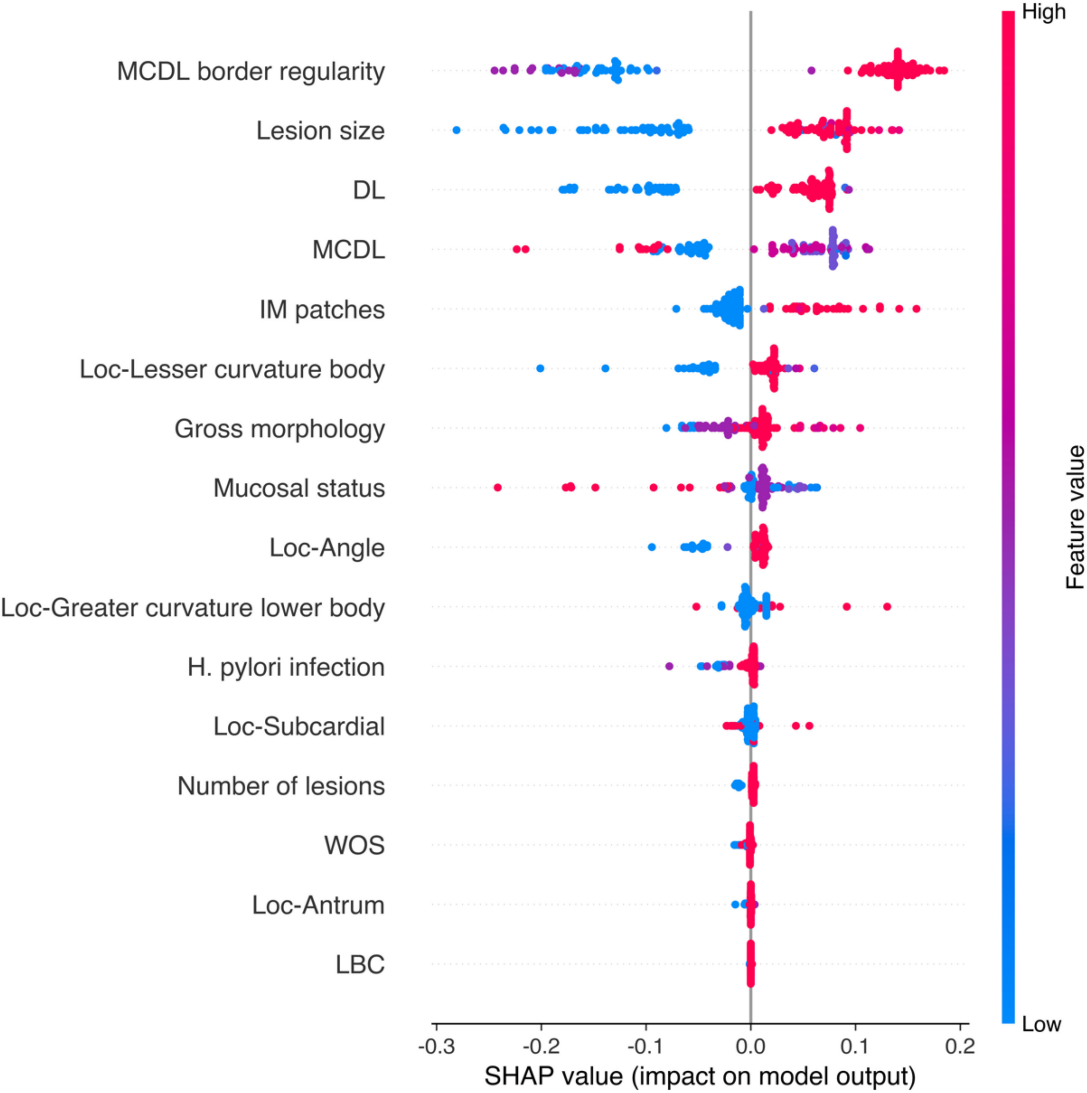
SHAP value scatter plot for the random forest OLGA staging diagnostic model.

**Figure 10:**
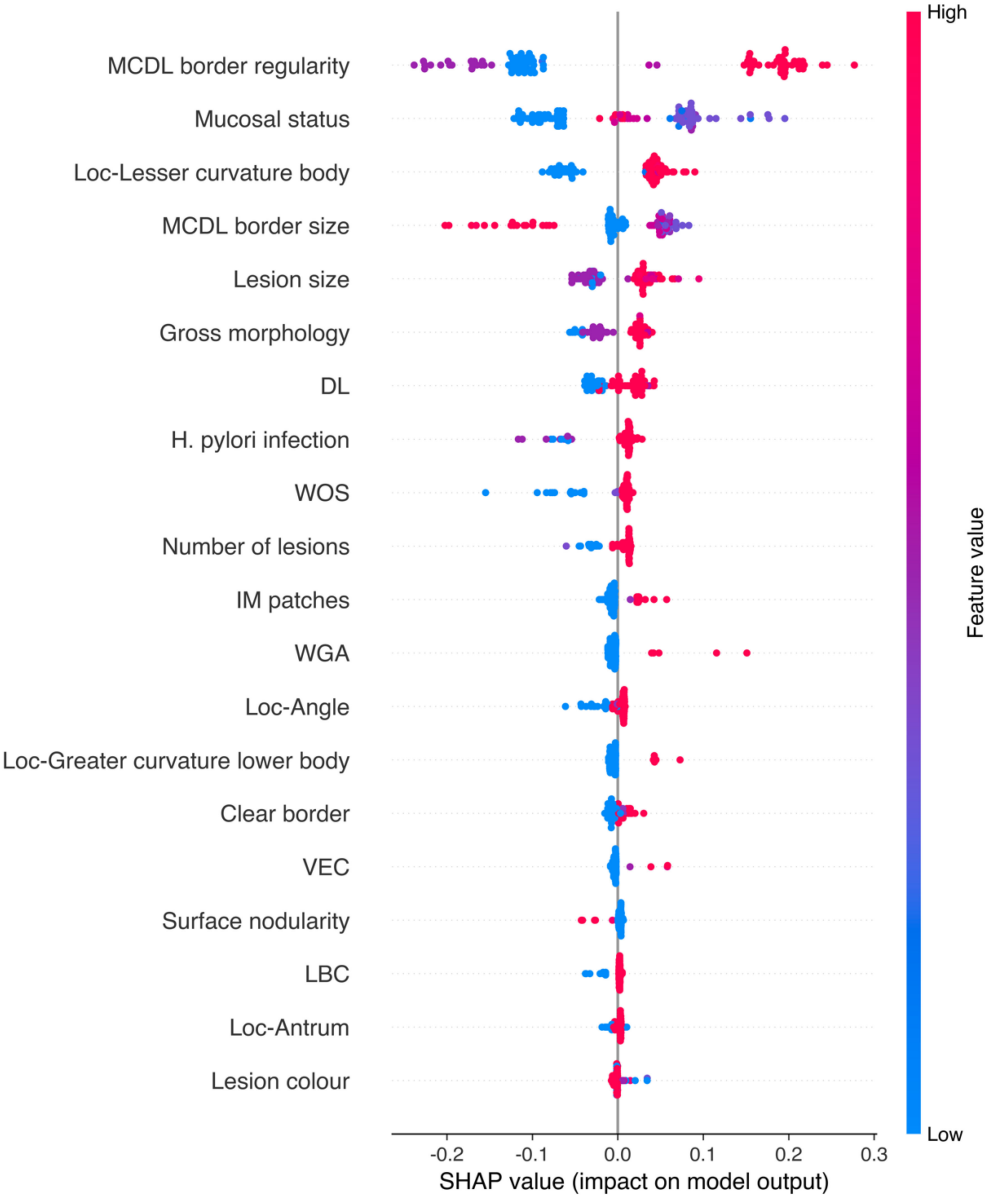
SHAP value scatter plot for the random forest OLGIM staging diagnostic model.

To gain deeper insight into the relationships between endoscopic features and model predictions, SHAP (SHapley Additive exPlanations) value analysis was performed to reveal the direction and magnitude of each feature’s impact on prediction outcomes (**Figures 9**, **10**). We found that certain features, such as MCDL border regularity and lesion size, demonstrated strong positive predictive effects in both OLGA and OLGIM diagnostic models.

### 3.7 Construction and validation of OLGA/OLGIM prediction models based on XGBoost

#### 3.7.1 Model construction

XGBoost algorithm was employed to construct diagnostic prediction models for OLGA and OLGIM staging. Following data standardisation, Synthetic Minority Over-sampling Technique (SMOTE) was applied to address class imbalance, ensuring comprehensive feature learning for both risk levels.

#### 3.7.2 Predictive model performance

**Figure 11:**
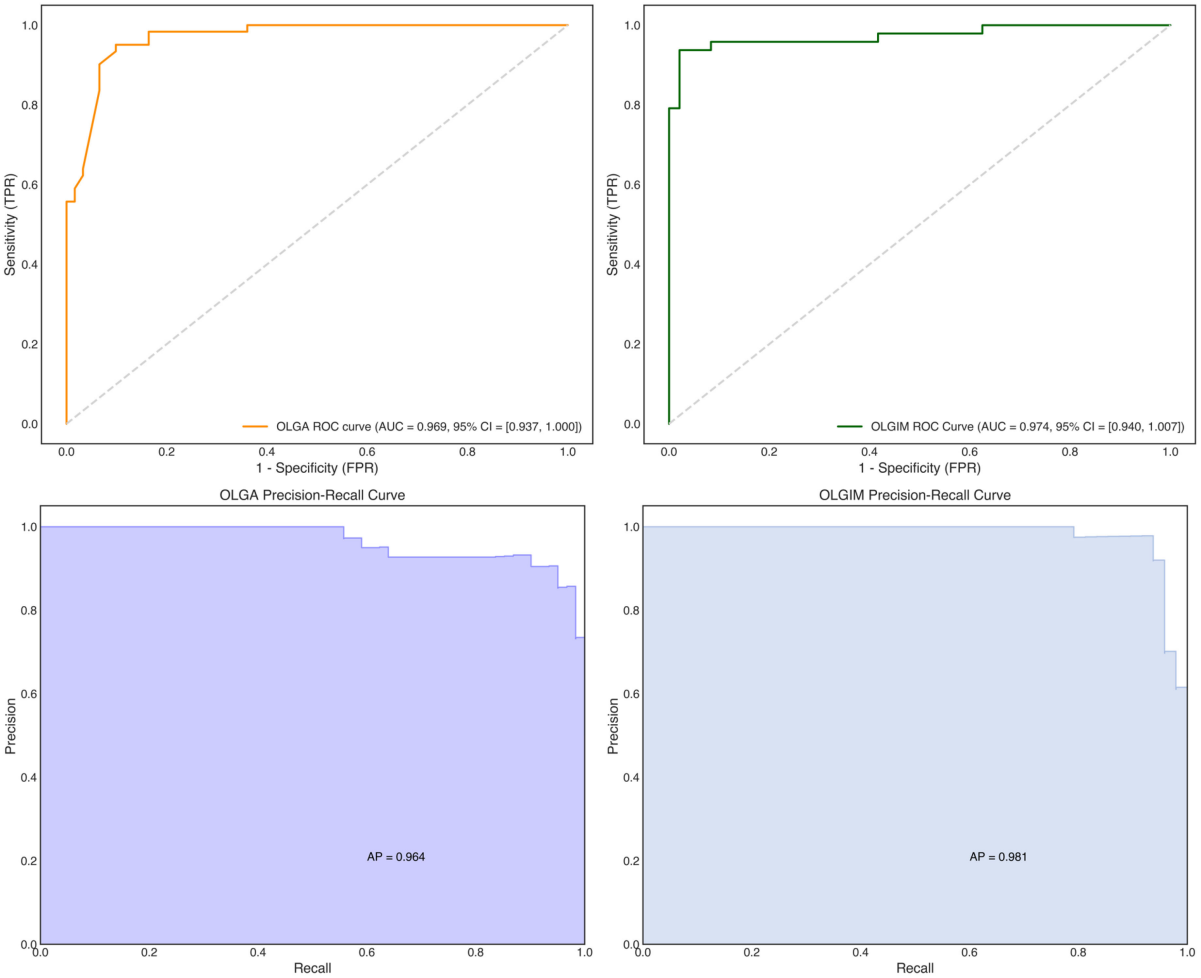
ROC and precision-recall curves for XGBoost OLGA and OLGIM staging diagnostic models.

The XGBoost models achieved accuracy rates of 0.918 and 0.927 for OLGA and OLGIM staging, respectively; specificity of 0.885 and 0.896; sensitivity of 0.951 and 0.958; precision of 0.892 and 0.902; AUC values of 0.966 and 0.979; and F1 scores of 0.921 and 0.929. Detailed performance metrics for both staging diagnostic models are presented in **Table 9**, with ROC curves shown in **Figure 11**. The XGBoost models demonstrated robust predictive capability for both OLGA and OLGIM staging, effectively discriminating between low-risk and high-risk OLGA/OLGIM patients.

**Table 9:** Performance of XGBoost OLGA and OLGIM staging diagnostic models.

Model	Accuracy	AUC	Specificity	Sensitivity	Precision	F1 Score
OLGA Model	0.918	0.966	0.885	0.951	0.892	0.921
OLGIM Model	0.927	0.979	0.896	0.958	0.902	0.929

Among the three models evaluated, XGBoost and Random Forest both demonstrated exceptional performance, with XGBoost exhibiting marginally superior metrics. As illustrated in **Table 9**, XGBoost achieved the highest accuracy (OLGA: 91.8%, OLGIM: 92.7%) and AUC values (OLGA: 0.966, OLGIM: 0.979). Random Forest also yielded excellent results, with accuracy (OLGA: 90.2%, OLGIM: 93.8%) and AUC values (OLGA: 0.958, OLGIM: 0.975) that were comparable to those of XGBoost. Given their high accuracy and AUC values, both XGBoost and Random Forest models show considerable promise for clinical application in risk stratification of precancerous gastric lesions. Whilst XGBoost demonstrates a slight advantage in performance metrics, the robust performance of Random Forest warrants acknowledgement. Therefore, based on a comprehensive evaluation of accuracy, AUC and overall robustness, we propose that both XGBoost and Random Forest models represent excellent candidates for real-world clinical implementation, with XGBoost potentially offering slight advantages due to its marginally superior performance.

To comprehensively evaluate clinical utility, we analysed both false positive (FP) and false negative (FN) outcomes across all models. In OLGA staging, the Bayesian model demonstrated notably high rates, with 48 false positives and 3 false negatives. Similarly, for OLGIM staging, the Bayesian model produced 49 false positives and 3 false negatives. The Random Forest model, however, exhibited markedly improved performance in OLGA staging, reducing false positives to 10 whilst maintaining only 2 false negatives. When applied to OLGIM staging, the Random Forest model yielded 3 false positives and 3 false negatives. The XGBoost model demonstrated promising results, with 7 false positives and 3 false negatives in OLGA staging, whilst in OLGIM staging, it produced 5 false positives and merely 2 false negatives. Notably, whilst the Random Forest model achieved optimal performance in OLGA staging with the lowest false negative count, the XGBoost model demonstrated superior overall balance across both staging systems, particularly in OLGIM staging where it combined minimal false negatives with relatively few false positives.

In the clinical management of precancerous gastric lesions, minimising false negatives is crucial, as failing to identify high-risk individuals requiring prompt intervention may result in delayed diagnosis and potential disease progression. Whilst less critical than false negatives, elevated false positive rates warrant consideration, as they may lead to unnecessary endoscopic procedures and biopsies in low-risk patients, thereby increasing healthcare expenditure and patient anxiety (26).

**Table 9** details the performance metrics of the XGBoost models. Both OLGA and OLGIM models achieved high accuracy (~0.92 and ~0.93 respectively) and AUC values (~0.966 and ~0.979 respectively), with high sensitivity and specificity, demonstrating robust and slightly superior performance compared to Random Forest and Bayesian models for risk prediction using XGBoost.

The XGBoost models exhibited exceptional performance in identifying high-risk cases, yielding F1-scores of 0.921 (OLGA) and 0.929 (OLGIM). **Figure 11** illustrates the corresponding Precision-Recall curves, providing detailed insights into the precision and recall dynamics for high-risk cohorts.

#### 3.7.3 Key predictive feature analysis

XGBoost algorithm was used to rank the importance of key predictive features in OLGA and OLGIM staging, with higher scores indicating greater diagnostic significance and contribution to predictive accuracy. Feature importance rankings and specific scores are shown in **Supplementary Figure 3** and **4**. These findings provide a basis for clinical focus on endoscopic features with higher numerical values in identifying high-risk precancerous gastric lesions.

**Figure 12:**
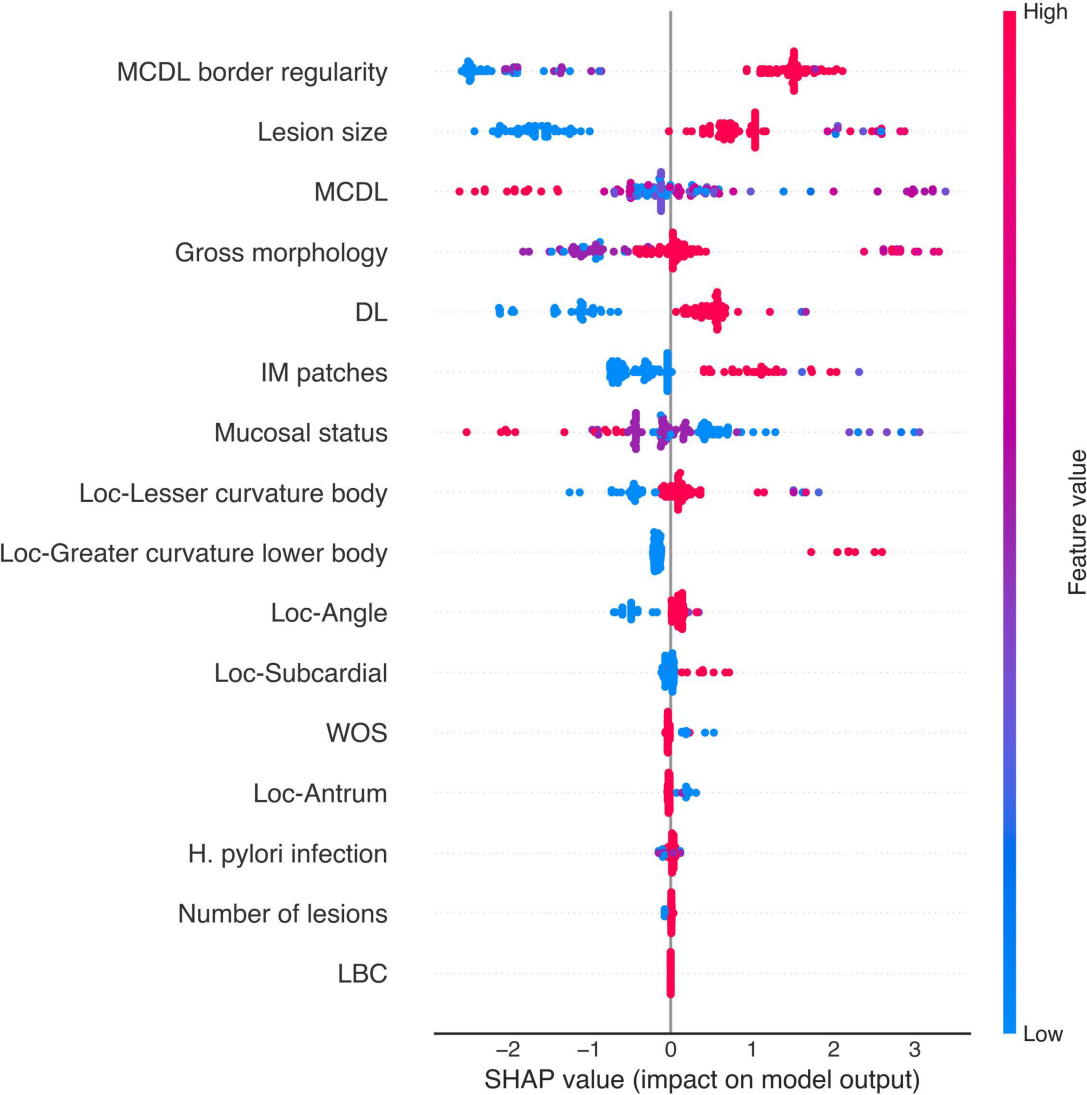
SHAP value scatter plot for the XGBoost OLGA staging diagnostic model.

**Figure 13:**
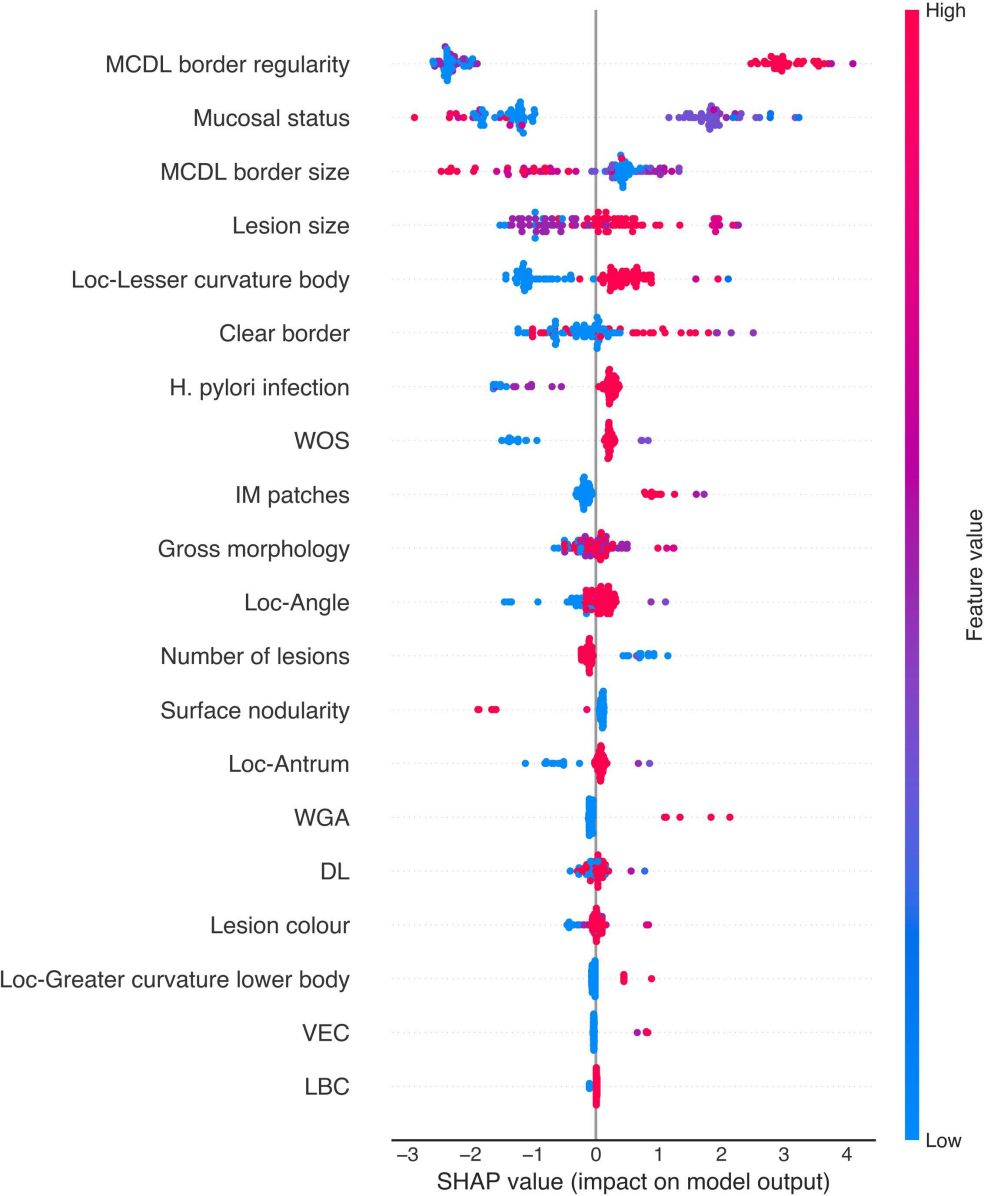
SHAP value scatter plot for the XGBoost OLGIM staging diagnostic model.

SHAP scatter plots for the XGBoost models are presented in **Figures 12** and **13**. In both OLGA and OLGIM staging diagnostic models, MCDL border irregularity and lesion size demonstrated strong positive predictive effects, whilst MCDL border size showed negative predictive effects. These results indicate that irregular MCDL and lesions ≥1cm are risk factors for high-risk OLGA/OLGIM, whereas larger MCDL border size serves as a protective factor for high-risk OLGA/OLGIM patients.

## 4 Discussion

Chronic inflammation induced by *H. pylori* infection can damage cells and promote gastric carcinogenesis through abnormal immune cell activation and increased inflammatory cytokine levels, making it a representative aetiological factor for gastric cancer (27). The progression from chronic gastritis to gastric cancer follows Correa’s cascade (28), where persistent inflammation drives progression towards gastric cancer, with CAG, IM, and Dys carrying risks for malignant transformation. Therefore, early screening and risk assessment of patients with potential for gastric cancer development remains an urgent clinical challenge. The OLGA and OLGIM systems provide a basis for predicting gastric cancer risk associated with atrophic gastritis and intestinal metaplasia, guiding clinical surveillance. ME-NBI clearly visualises superficial mucosal and vascular patterns, with studies showing sensitivity of 88% and specificity of 96% in distinguishing cancerous from non-cancerous lesions (29). The combination of C-WLI and ME-NBI enhances gastric cancer detection rates, laying the foundation for “endoscopic pathology”.

In this study, we employed three models: the linear Bayesian stepwise discrimination analysis and two non-linear models (random forest and XGBoost), to construct OLGA and OLGIM staging prediction models based on C-WLI and ME-NBI endoscopic features. The comparative performance metrics of these models are summarised in **Table 10**.

**Table 10:** Summary of specific performance of the three models.

	OLGA			OLGIM		
	Bayesian Stepwise Discrimination	Random Forest	XGBoost	Bayesian Stepwise Discrimination	Random Forest	XGBoost
Accuracy	0.854	0.902	0.918	0.910	0.938	0.927
AUC	0.928	0.958	0.966	0.924	0.975	0.979
Specificity	0.842	0.836	0.885	0.839	0.938	0.896
Sensitivity	0.942	0.967	0.951	0.942	0.938	0.958
Precision	0.505	0.855	0.892	0.500	0.938	0.902

In the Bayesian stepwise discrimination model, we employed stepwise variable selection to eliminate redundant variables, simplifying model structure and enhancing predictive accuracy, successfully constructing linear regression equations for OLGA and OLGIM staging (see Results section 3.5.1). This method’s advantage lies in producing intuitive linear regression equations with good interpretability. The Bayesian models demonstrated high predictive accuracy (0.854 and 0.910) and sensitivity (both 0.942) for OLGA and OLGIM staging. However, their relatively low precision (0.505 and 0.500) indicates that whilst the models effectively avoid false negatives, approximately half of low-risk patients were misclassified as high-risk, risking over-treatment. We attribute this to the complex diversity of C-WLI and ME-NBI endoscopic features and their potentially complex non-linear relationships with OLGA and OLGIM staging. Consequently, we employed random forest and XGBoost non-linear models for further analysis.

As shown in **Table 10**, the random forest algorithm achieved excellent results in OLGA and OLGIM staging prediction, with accuracy rates of 0.902 and 0.938, and sensitivity of 0.967 and 0.938, respectively. Precision improved significantly compared to the Bayesian model, reaching 0.855 and 0.938. These results further confirm the complex non-linear relationships between C-WLI and ME-NBI endoscopic features and OLGA/OLGIM staging. XGBoost, an emerging gradient boosting method, excels in handling complex non-linear relationships and data imbalance (30, 31). Its predictive performance for OLGA and OLGIM staging was comparable to the random forest model, achieving accuracy rates of 0.918 and 0.927, sensitivity of 0.951 and 0.958, and precision of 0.892 and 0.902, significantly outperforming the Bayesian model.

Overall, all three models demonstrated exceptionally high accuracy and sensitivity, but with notable differences in precision. Whilst all models effectively reduce the risk of missed diagnoses, random forest and XGBoost models show superior clinical utility by significantly reducing over-diagnosis risk compared to the Bayesian model. Each model offers unique advantages: Bayesian stepwise discrimination excels in model interpretability, random forest in feature selection, and XGBoost in capturing complex variable relationships. Together, these models provide reliable theoretical foundations for clinical application.

The primary clinical utility of our models resides in their application as a triage instrument during endoscopic evaluation. Whilst not designed to supplant histopathological examination for definitive OLGA/OLGIM staging, incorporation of these models into real-time endoscopic systems may assist clinicians by providing immediate risk stratification. This integration could facilitate targeted sampling of high-risk lesions, potentially enhancing diagnostic yield and procedural efficiency, whilst supporting contemporaneous clinical decision-making regarding patient management and surveillance protocols.

Our investigation encompasses several notable limitations. Principally, the single-centre nature of the study necessitates multi-centre validation to establish broader generalisability. Additionally, external validation utilising independent datasets is essential to further substantiate model robustness. Furthermore, real-time clinical evaluation is requisite to assess the practical applicability of these models during live endoscopic procedures.

Whilst the sample size (n=356) is relatively modest for complex machine learning models, particularly considering the inherent class imbalance in OLGA/OLGIM staging, it remains comparable to analogous investigations in AI-assisted endoscopic diagnosis (19, 20). We implemented SMOTE methodology to address this imbalance specifically, thereby augmenting the minority class (high-risk stages) effectively. The robust performance metrics of our models, notably the elevated sensitivity in detecting high-risk cases (exceeding 0.94 across all models), suggest that the sample size, in conjunction with SMOTE augmentation, proved sufficient for this exploratory investigation. Further validation through larger, multi-centre studies is scheduled for subsequent research.

The exclusion criteria (e.g., autoimmune gastritis, ulcers) may constrain the model’s generalisability. Whilst necessary for internal validity, these exclusions indicate that the model is primarily validated for patients without these comorbidities. Future research should evaluate the model’s performance in more diverse populations to enhance its clinical utility.

Another significant limitation warranting acknowledgement is this study’s dependence on histopathology as the reference standard for OLGA/OLGIM staging, which itself demonstrates considerable inter-observer variability. This inherent inconsistency in the reference standard introduces an element of uncertainty into our model development and validation, potentially influencing the absolute accuracy metrics. This limitation underscores the necessity for subsequent investigations to explore more objective diagnostic parameters or to quantify and address this variability explicitly in methodological approaches.

Notwithstanding these constraints, our models demonstrate considerable potential for clinical application. Integration into AI-enhanced endoscopy systems could facilitate real-time OLGA/OLGIM risk stratification, thereby augmenting gastroenterologists’ decision-making processes during procedures. However, it is imperative to emphasise that these models should serve as complementary tools rather than substitutes for clinical judgment.

Regarding clinical implementation, model selection warrants careful consideration of the balance between interpretability and performance metrics. The Bayesian model offers superior interpretability, albeit with reduced precision. Conversely, Random Forest and XGBoost algorithms deliver enhanced accuracy, with XGBoost demonstrating marginally superior performance in our investigation. Random Forest exhibits particular robustness in feature selection, whilst XGBoost demonstrates exceptional capability in processing complex datasets. Clinicians may opt for the Bayesian model when transparency is paramount, or select machine learning models when heightened accuracy is essential, contingent upon specific clinical contexts and priorities. Comprehensive validation studies remain necessary to inform optimal model selection and implementation strategies.

Risk factors for high-risk OLGA staging across the three models are shown in **Table 11**. Extensive gastric mucosal atrophy represents a crucial risk factor for intestinal-type gastric cancer (32). The OLGA staging system encompasses both the severity and extent of atrophy, making it suitable for evaluating atrophic gastritis (33). Kimura and Takemoto classified atrophy based on endoscopic atrophic border appearance into closed and open types. O-1 represents moderate atrophy, whilst O-2 and O-3 indicate severe atrophy; O-1 extends beyond the cardia, O-2 reaches the gastric fundus, and O-3 extends to the greater curvature of the lower gastric body (34). In our study, lesions in the subcardial region and greater curvature of the lower gastric body corresponded to moderate-severe atrophy distribution, thus being incorporated as influential factors in the high-risk OLGA diagnostic model. The severity and extent of IM similarly serve as crucial indicators for predicting gastric cancer risk. Research indicates that patients with concurrent *H. pylori* infection and IM face a 6.4-fold higher gastric cancer risk compared to those with *H. pylori* infection alone (3). Whilst IM diagnosis primarily relies on histological assessment, our study found that some patients exhibited flat or slightly elevated white patches under C-WLE (**Figure 2A**). ME-NBI examination of these white patches revealed characteristic LBC (**Figure 2B**). Given LBC’s close association with gastric cancer development and diagnosis, we incorporated intestinal metaplasia patches observed under C-WLE into the high-risk OLGA diagnostic model.

**Table 11:** Risk factors for high-risk OLGA.

Model	High-risk OLGA risk factors				
Bayesian Stepwise Discrimination	Subcardial	Greater curvature of the lower gastric body	IM patches	Lesion size	MCDL border regularity
Random Forest	MCDL border regularity	MCDL border size	DL	Lesion size	Gross morphology
XGBoost	MCDL border regularity	DL	Greater curvature of the lower gastric body	MCDL border size	Lesion size

The demarcation line (DL) manifests as a distinct boundary between lesional and non-lesional regions (35). Yao et al., in their characterisation of endoscopic features of gastric atrophy, provided detailed observations of microvascular and micro-glandular structural alterations under ME-NBI examination within the context of atrophic gastritis. These alterations culminate in the formation of demarcation lines, establishing DL as an endoscopic indicator of atrophy and thereby suggesting their intrinsic association. Multiple convex demarcation line (MCDL) represents a more refined manifestation of DL, presenting as multiple convex demarcation boundaries along the surrounding mucosal epithelial margin. Under ME-NBI examination, early gastric carcinoma exhibits distinct microvascular morphological characteristics compared to the surrounding non-neoplastic regions (typically atrophic gastritis). The microvascular alterations observed in atrophic gastritis form the fundamental basis for demarcation line formation, including MCDL. The regularity of these boundaries holds particular significance in determining the malignant potential of lesions. Studies have shown that MCDL border regularity demonstrates sensitivity, specificity, and precision (positive predictive value) of 38%, 91%, and 97%, respectively, for non-cancerous lesions (36). In our Bayesian discrimination analysis, MCDL border regularity was incorporated into the high-risk OLGA diagnostic model with a substantial variable coefficient of 5.696. This indicates that irregular MCDL borders correlate with higher malignant transformation risk, consistent with previous research findings. In random forest and XGBoost analyses, MCDL border size showed negative predictive value for gastric cancer risk. This finding aligns with previous studies suggesting MCDL border size as a predictor of non-cancerous lesions. Research proposes a threshold of two-thirds for MCDL border size in distinguishing non-cancerous from cancerous lesions, with MCDL border size ≥2/3 considered a protective factor (37), highly consistent with our findings.

Risk factors for high-risk OLGIM staging across the three models are shown in **Table 12**. The high-risk OLGIM diagnostic model considers lesions not only in the greater curvature of the lower gastric body but also specifically in the lesser curvature. Research indicates that IM occurring in the lesser curvature carries a higher risk of gastric cancer development (38), supporting our inclusion of lesser curvature IM as a predictive factor for high-risk OLGIM. VEC refers to characteristic vessels within epithelial circles surrounded by circular marginal crypt epithelium (**Figure 2C**). Studies suggest that the VEC pattern may characterise papillary adenocarcinoma, serving as an effective preoperative marker for high-grade malignancy (39). Our incorporation of VEC into the high-risk OLGIM diagnostic model confirms its value as an effective endoscopic predictor. WGA appears as small white globular structures beneath the gastric epithelium. Histopathologically, WGA represents dilated gland lumina containing eosinophilic material with necrotic epithelial fragments (40). A prospective study of WGA showed incidence rates of 21.4% and 2.5% in cancerous and non-cancerous lesions respectively (P<0.001), with accuracy, sensitivity, and specificity of 69.1%, 21.4%, and 97.5% for gastric cancer detection (41). Among our 365 patients, 14 (3.9%) exhibited WGA. In the Bayesian analysis high-risk OLGIM model equation, WGA’s coefficient of 2.888 confirms its significance as a predictive indicator.

**Table 12:** Risk factors for high-risk OLGIM.

Model	High-risk OLGIM risk factors				
Bayesian Stepwise Discrimination	*H. pylori* infection	Lesser curvature of the gastric body	Greater curvature of the lower gastric body	Erosion	Lesion size
Random Forest	MCDL border regularity	Mucosal status	MCDL border size	Lesser curvature of the gastric body	Gross morphology
XGBoost	Greater curvature of the lower gastric body	MCDL border regularity	WGA	Mucosal status	Surface nodularity

Our statistics show that among high-risk OLGIM patients, 96.6% exhibited past *H. pylori* infection status, and 76.7% showed map-like and patchy redness, typical manifestations of past *H. pylori* infection (21). Previous research indicates that map-like redness serves not only as an independent factor for high-risk OLGA/OLGIM staging (42) but also as an independent risk factor for gastric cancer development after *H. pylori* eradication (43). Based on these findings, we incorporated *H. pylori* infection status as a significant risk factor in the high-risk OLGIM diagnostic model.

Furthermore, to control for *H. pylori* infection as a potential confounder and remove its influence on the results, we included *H. pylori* infection status as an independent variable in all analytical models (Bayesian stepwise discrimination, Random Forest, and XGBoost). Through this methodological approach, we were able to adjust for the influence of *H. pylori* infection during statistical analyses, whether through calculating the independent effects of individual variables on OLGIM staging in multivariate regression models, or through capturing complex relationships and interactions among various features in machine learning models. This systematic adjustment enabled more precise evaluation of the independent associations between endoscopic features and OLGIM staging classifications.

## 5 Conclusion

In this study, we developed and validated diagnostic models for OLGA and OLGIM staging of precancerous gastric lesions using endoscopic features. Our findings demonstrate that machine learning approaches, particularly random forest and XGBoost algorithms, can effectively predict high-risk OLGA/OLGIM stages based on conventional white-light endoscopy and magnifying endoscopy with narrow-band imaging features. These models achieved high accuracy (>90%) and AUC values (>0.95), outperforming traditional Bayesian stepwise discrimination analysis.

Key endoscopic risk factors for high-risk OLGA/OLGIM stages included irregular multiple convex demarcation line borders, lesion size ≥1cm, and specific lesion locations such as the greater curvature of the lower gastric body. Additionally, features like white globe appearance and vessel within epithelial circle patterns emerged as significant predictors for high-risk OLGIM. The identification of these risk factors provides valuable insights into the endoscopic characteristics associated with advanced precancerous lesions.

These findings have important clinical implications, potentially enabling more accurate risk stratification of patients with precancerous gastric lesions using readily available endoscopic techniques. This could lead to more targeted surveillance strategies and earlier interventions for high-risk individuals, potentially improving the early detection and management of gastric cancer. The ability to predict OLGA/OLGIM stages based on endoscopic features may also reduce the need for extensive biopsy sampling in some cases, streamlining the diagnostic process.

However, our study has limitations that should be addressed in future research. Notably, this was a single-centre study with internal validation only. To establish the generalizability and robustness of these models, multi-centre studies with larger, diverse patient populations are needed. Additionally, prospective studies evaluating the clinical impact of implementing these models in real-world settings would be valuable.

In conclusion, our results suggest that machine learning-based analysis of endoscopic features can provide valuable diagnostic and prognostic information for precancerous gastric lesions. This approach may complement histopathological assessment and enhance risk prediction in clinical practice. By combining advanced imaging techniques with sophisticated analytical tools, we may be able to improve the accuracy and efficiency of gastric cancer risk assessment. Future research should focus on external validation, refinement of these models, and assessment of their impact on clinical outcomes to fully realize their potential in improving gastric cancer prevention and early detection strategies.

## Data Availability

The original contributions presented in the study are included in the article/**Supplementary Material**. Further inquiries can be directed to the corresponding author.
