# Supplementary figures and images for: Risk prediction model for precancerous gastric lesions based on magnifying endoscopy combined with narrow-band imaging features
Source: Front Oncol. 2025 Apr 4;15:1554523. doi: 10.3389/fonc.2025.1554523 (PMC12006015; doi:10.3389/fonc.2025.1554523)

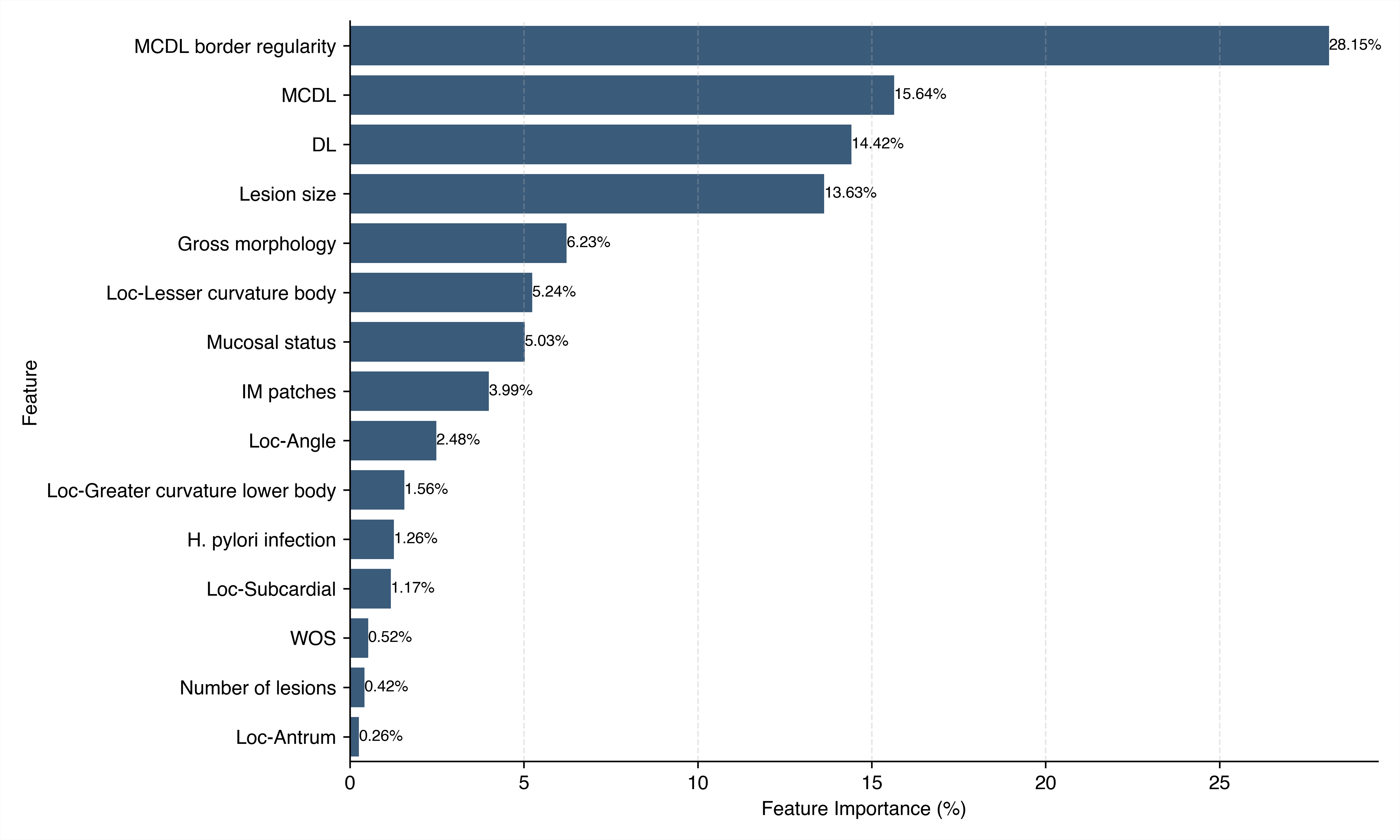

Supplement: Supplementary file 1 [file Image1.tiff]

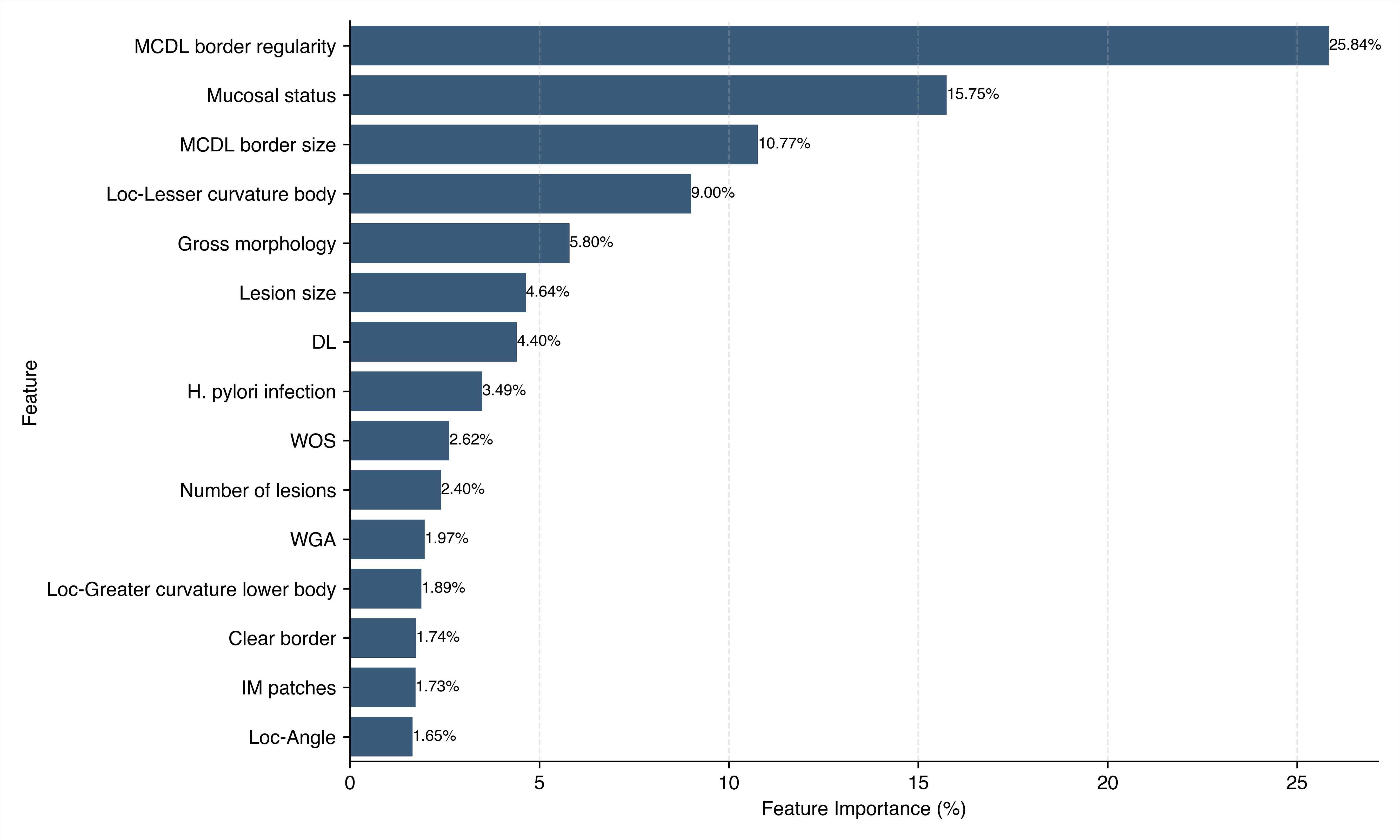

Supplement: Supplementary file 2 [file Image2.tiff]

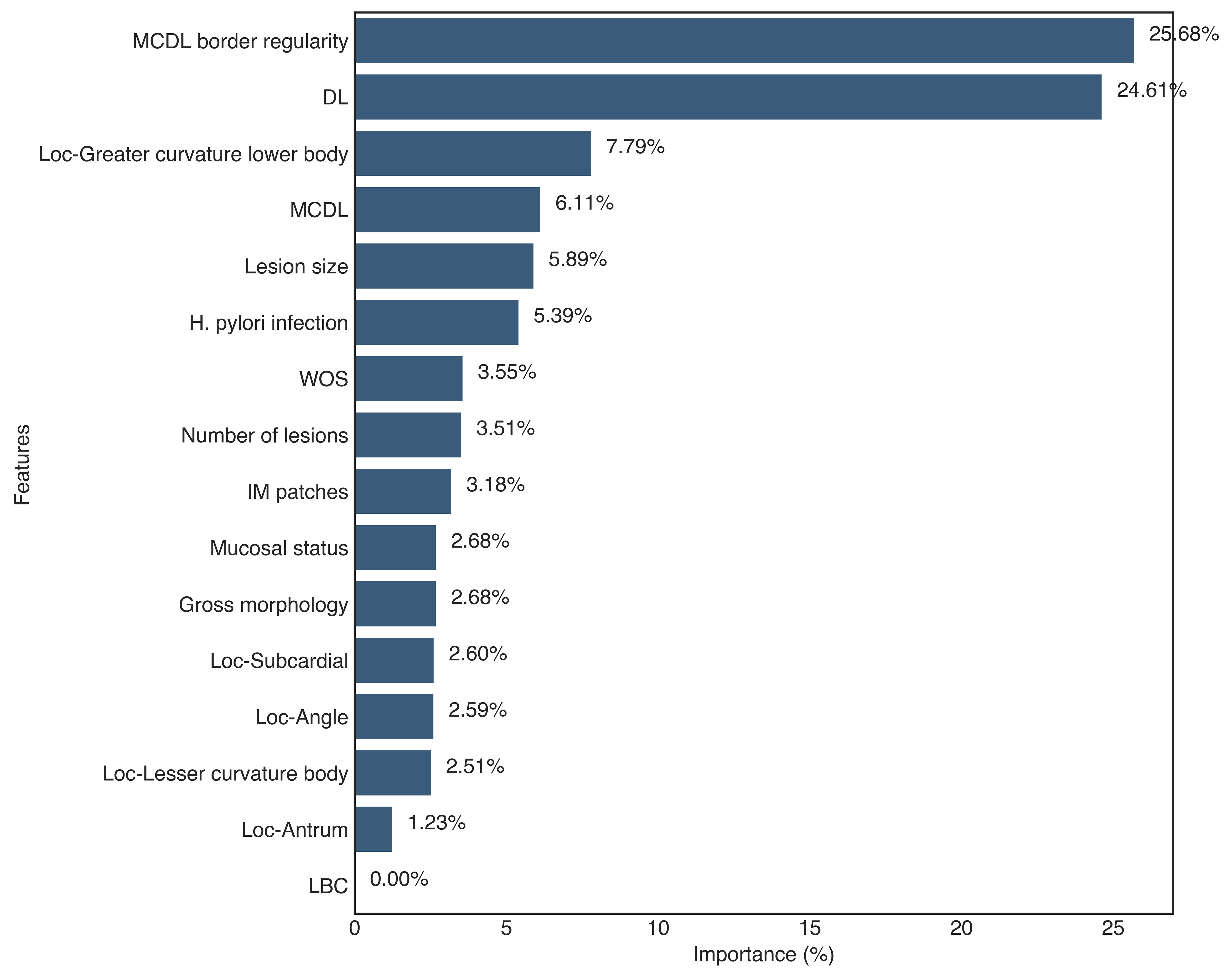

Supplement: Supplementary file 3 [file Image3.tiff]

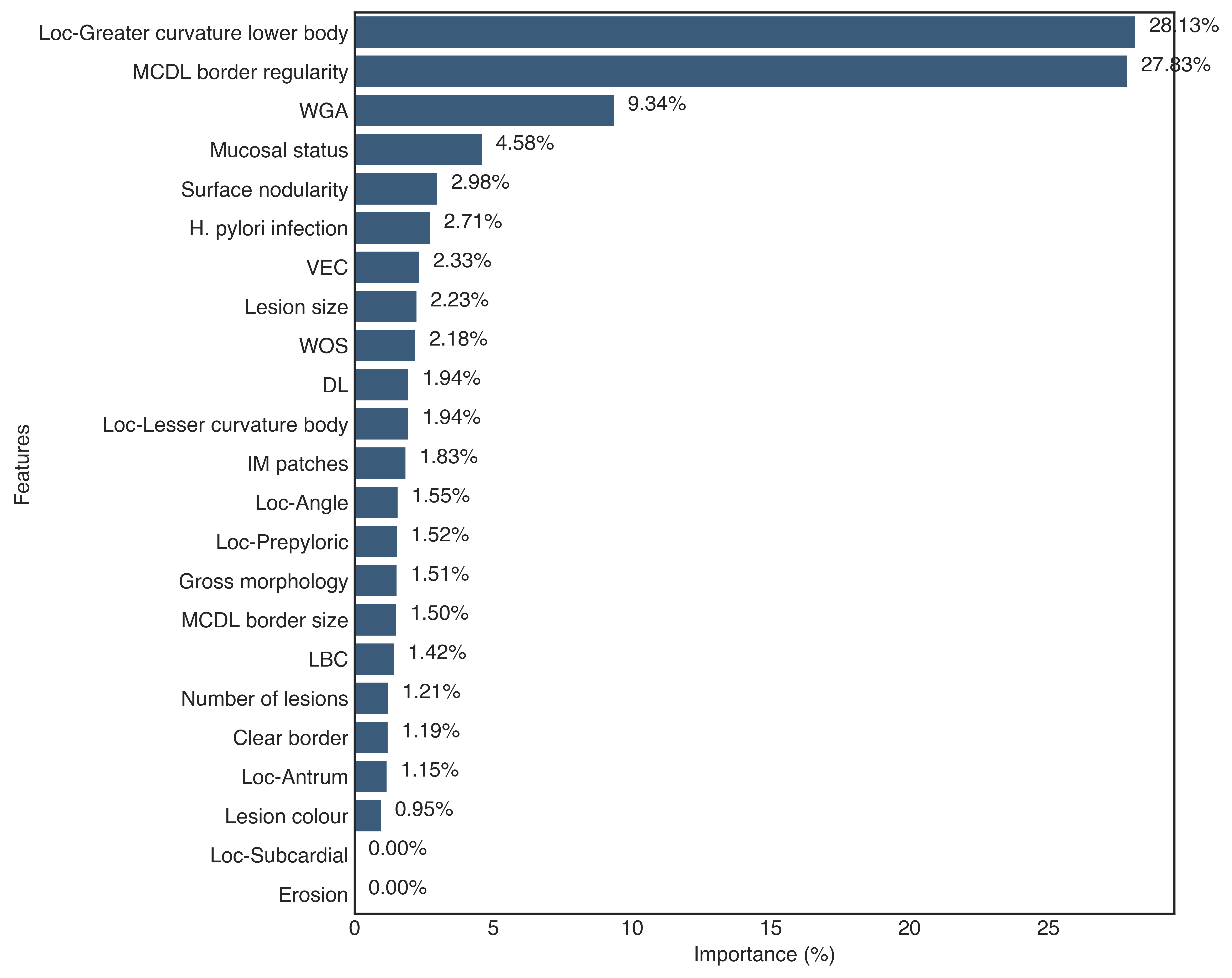

Supplement: Supplementary file 4 [file Image4.tiff]
