# Supplementary material for: Risk prediction model for precancerous gastric lesions based on magnifying endoscopy combined with narrow-band imaging features
Source: Front Oncol. 2025 Apr 4;15:1554523. doi: 10.3389/fonc.2025.1554523 (PMC12006015; doi:10.3389/fonc.2025.1554523)
Supplement: Supplementary file 5 [file Table1.docx]

***Supplementary Material***

**Risk Prediction Model for Precancerous Gastric Lesions Based on Magnifying Endoscopy Combined with Narrow-band Imaging Features**

**Supplementary Table**

Supplementary TABLE 1. Demographic Characteristics Based on OLGA/OLGIM Staging

|  | Low-risk OLGA/OLGIM | High-risk OLGA/OLGIM | Total OLGA/OLGIM | P-value （OLGA/OLGIM） |
| --- | --- | --- | --- | --- |
| Sex |  |  |  | 0.251/0.066 |
| Female | 143/118 | 20/45 | 163 |  |
| Male | 161/122 | 32/71 | 193 |  |
| Age |  |  |  | 0.164/0.196 |
| ≤40 | 18/17 | 1/2 | 19 |  |
| 41-50 | 61/45 | 6/22 | 67 |  |
| 51-60 | 117/90 | 19/46 | 136 |  |
| ＞60 | 108/88 | 26/46 | 134 |  |

Table 1 presents the demographic characteristics of the study cohort stratified by OLGA and OLGIM risk categories. No statistically significant differences in age or sex distribution were observed between low-risk and high-risk groups for either staging system (*P* > 0.05). This indicates demographic homogeneity across risk strata, suggesting these factors are unlikely to confound the analysis of endoscopic features.
