# Supplementary material for: Risk prediction model for precancerous gastric lesions based on magnifying endoscopy combined with narrow-band imaging features
Source: Front Oncol. 2025 Apr 4;15:1554523. doi: 10.3389/fonc.2025.1554523 (PMC12006015; doi:10.3389/fonc.2025.1554523)
Supplement: Supplementary file 6 [file Table2.docx]

***Supplementary Material***

**Risk Prediction Model for Precancerous Gastric Lesions Based on Magnifying Endoscopy Combined with Narrow-band Imaging Features**

**Supplementary Table**

Supplementary TABLE 2. Lesion Characteristics Based on OLGA Staging (C-WLE findings)

| Characteristic | Low-risk OLGA | High-risk OLGA | Total | P-value |
| --- | --- | --- | --- | --- |
| *H. pylori* infection |  |  |  | ＜0.001^*^ |
| None | 48（15.8%） | 0（0.0%） | 48 |  |
| Current | 29（9.5%） | 2（3.8%） | 31 |  |
| Past | 227（74.7%） | 50（96.2%） | 277 |  |
| Mucosal status |  |  |  | ＜0.001^*^ |
| None | 192（63.2%） | 15（28.8%） | 207 |  |
| Map-like redness | 55（18.1%） | 36（69.2%） | 91 |  |
| Patchy redness | 40（13.2%） | 0（0.0%） | 40 |  |
| Chicken skin appearance | 1（0.3%） | 0（0.0%） | 1 |  |
| Diffuse redness | 12（3.9%） | 1（1.9%） | 13 |  |
| Mucosal oedema | 4（1.3%） | 0（0.0%） | 4 |  |
| Number of lesions |  |  |  | ＜0.001^*^ |
| None | 13（4.3%） | 0（0.0%） | 13 |  |
| Single | 72（23.7%） | 0（0.0%） | 72 |  |
| Multiple | 219（72.0%） | 52（100.0%） | 271 |  |
| Location |  |  |  |  |
| Subcardial | 13（2.1%） | 9（5.6%） | 22 |  |
| Lesser curvature of body | 134（21.3%） | 44（27.5%） | 178 |  |
| Gastric Fundus | 4（0.6%） | 0（0.0%） | 4 |  |
| Gastric Angle | 199（31.6%） | 48（30.0%） | 247 |  |
| Gastric Antrum | 242（38.5%） | 50（31.2%） | 292 |  |
| Anterior and posterior walls of the upper gastric body | 3（0.5%） | 0（0.0%） | 3 |  |
| Greater curvature of the lower gastric body | 4（0.6%） | 8（5.0%） | 12 |  |
| Pre-pyloric region | 30（4.8%） | 1（0.6%） | 31 |  |
| Lesion size |  |  |  | ＜0.001^*^ |
| 0 | 20（6.6%） | 0（0.0%） | 20 |  |
| <1 cm | 182（59.9%） | 13（25.0%） | 195 |  |
| ≥1 cm | 102（33.6%） | 39（75.0%） | 141 |  |
| Morphology |  |  |  | ＜0.001^*^ |
| None | 13（4.3%） | 0（0.0%） | 13 |  |
| Elevated | 37（12.2%） | 1（1.9%） | 38 |  |
| Flat | 140（46.1%） | 12（23.1%） | 152 |  |
| Depressed | 114（37.5%） | 39（75.0%） | 153 |  |
| Colour |  |  |  | 0.517 |
| None | 13（4.3%） | 0（0.0%） | 13 |  |
| Same | 13（4.3%） | 1（1.9%） | 14 |  |
| Pale | 51（16.8%） | 9（17.3%） | 60 |  |
| Red | 227（74.7%） | 42（80.8%） | 269 |  |
| Erosion |  |  |  | 0.223 |
| Absent | 283（93.1%） | 51（98.1%） | 334 |  |
| Present | 21（6.9%） | 1（1.9%） | 22 |  |
| Surface nodularity |  |  |  | 0.864 |
| Absent | 277（91.1%） | 47（90.4%） | 324 |  |
| Present | 27（8.9%） | 5（9.6%） | 32 |  |
| Ulceration | |  |  | 1.000 |
| Absent | 302（99.3%） | 52（100.0%） | 354 |  |
| Present | 2（0.7%） | 0（0.0%） | 2 |  |
| Clear border | |  |  | 0.079 |
| Absent | 218（71.7%） | 31（59.6%） | 249 |  |
| Present | 86（28.3%） | 21（40.4%） | 107 |  |
| IM patches | |  |  | ＜0.001^*^ |
| Absent | 283（93.1%） | 34（65.4%） | 317 |  |
| Present | 21（6.9%） | 18（34.6%） | 39 |  |

Note: **P* < 0.05

Table 2 compares C-WLE lesion characteristics between OLGA stages. Significant differences (*P* < 0.001) were found in *H. pylori* infection history, mucosal status, lesion number and size, morphology, IM patches. High-risk OLGA was associated with past *H. pylori*, map-like redness, multiple lesions, depressed morphology, IM patches, and larger size, highlighting macroscopically visible features indicative of advanced stages.
