# Supplementary material for: Risk prediction model for precancerous gastric lesions based on magnifying endoscopy combined with narrow-band imaging features
Source: Front Oncol. 2025 Apr 4;15:1554523. doi: 10.3389/fonc.2025.1554523 (PMC12006015; doi:10.3389/fonc.2025.1554523)
Supplement: Supplementary file 7 [file Table3.docx]

***Supplementary Material***

**Risk Prediction Model for Precancerous Gastric Lesions Based on Magnifying Endoscopy Combined with Narrow-band Imaging Features**

**Supplementary Table**

Supplementary TABLE 3. Lesion Characteristics Based on OLGA Staging (ME-NBI findings)

| Characteristic | Low-risk OLGA | High-risk OLGA | Total | P-value |
| --- | --- | --- | --- | --- |
| DL |  |  |  | ＜0.001^*^ |
| Absent | 180（59.2%） | 6（11.5%） | 186 |  |
| Present | 124（40.8%） | 46（88.5%） | 170 |  |
| IMVP |  |  |  | 0.598 |
| Absent | 275（90.5%） | 49（94.2%） | 324 |  |
| Present | 29（9.5%） | 3（5.8%） | 32 |  |
| IMSP |  |  |  | 1.000 |
| Absent | 277（91.1%） | 48（92.3%） | 325 |  |
| Present | 27（8.9%） | 4（7.7%） | 31 |  |
| LBC |  |  |  | 0.003^*^ |
| Absent | 37（12.2%） | 0（0.0%） | 37 |  |
| Present | 267（87.8%） | 52（100.0%） | 319 |  |
| WOS |  |  |  | 0.002^*^ |
| Absent | 75（24.7%） | 3（5.8%） | 78 |  |
| Present | 229（75.3%） | 49（94.2%） | 278 |  |
| WGA |  |  |  | 0.440 |
| Absent | 293（96.4%） | 49（94.2%） | 342 |  |
| Present | 11（3.6%） | 3（5.8%） | 14 |  |
| MCDL border size |  |  |  | ＜0.001^*^ |
| 0 | 179（58.9%） | 6（11.5%） | 185 |  |
| >0 to <1/3 | 22（7.2%） | 25（48.1%） | 47 |  |
| ≥1/3 to <2/3 | 29（9.5%） | 21（40.4%） | 50 |  |
| ≥2/3 | 74（24.3%） | 0（0.0%） | 74 |  |
| MCDL border regularity |  |  |  | ＜0.001^*^ |
| None | 180（59.2%） | 6（11.5%） | 186 |  |
| Regular | 78（25.7%） | 0（0.0%） | 78 |  |
| Irregular | 46（15.1%） | 46（88.5%） | 92 |  |
| VEC |  |  |  | 0.266 |
| Absent | 287（94.4%） | 47（90.4%） | 334 |  |
| Present | 17（5.6%） | 5（9.6%） | 22 |  |

Note: **P* < 0.05

Table 3 details ME-NBI features and OLGA stage. Significant differences (*P* < 0.001) were observed for DL, MCDL border regularity and size, and LBC and WOS presence (*P* ≤ 0.003). High-risk OLGA showed increased DL, LBC, WOS, irregular MCDL border, and larger MCDL size, demonstrating ME-NBI's ability to detect microstructural features associated with advanced OLGA.
