# Supplementary material for: Risk prediction model for precancerous gastric lesions based on magnifying endoscopy combined with narrow-band imaging features
Source: Front Oncol. 2025 Apr 4;15:1554523. doi: 10.3389/fonc.2025.1554523 (PMC12006015; doi:10.3389/fonc.2025.1554523)
Supplement: Supplementary file 8 [file Table4.docx]

***Supplementary Material***

**Risk Prediction Model for Precancerous Gastric Lesions Based on Magnifying Endoscopy Combined with Narrow-band Imaging Features**

**Supplementary Table**

Supplementary TABLE 4. Lesion Characteristics Based on OLGIM Staging (C-WLE findings)

| Characteristic | Low-risk OLGIM | High-risk OLGIM | Total | P-value |
| --- | --- | --- | --- | --- |
| *H. pylori* infection |  |  |  | ＜0.001^*^ |
| None | 48（20.0%） | 0（0.0%） | 48 |  |
| Current | 27（11.2%） | 4（3.4%） | 31 |  |
| Past | 165（68.8%） | 112（96.6%） | 277 |  |
| Mucosal status |  |  |  | ＜0.001^*^ |
| None | 182（75.8%） | 25（21.6%） | 207 |  |
| Map-like redness | 11（4.6%） | 80（69.0%） | 91 |  |
| Patchy redness | 31（12.9%） | 9（7.8%） | 40 |  |
| Chicken skin appearance | 1（0.4%） | 0（0.0%） | 1 |  |
| Diffuse redness | 12（5.0%） | 1（0.9%） | 13 |  |
| Mucosal oedema | 3（1.2%） | 1（0.9%） | 4 |  |
| Number of lesions |  |  |  | ＜0.001^*^ |
| None | 13（5.4%） | 0（0.0%） | 13 |  |
| Single | 69（28.7%） | 3（2.6%） | 72 |  |
| Multiple | 158（65.8%） | 113（97.4%） | 271 |  |
| Location |  |  |  |  |
| Subcardial | 9（2.0%） | 13（3.8%） | 22 |  |
| Lesser curvature of the gastric body | 81（18.1%） | 97（28.4%） | 178 |  |
| Gastric Fundus | 2（0.4%） | 2（0.6%） | 4 |  |
| Gastric Angle | 144（32.1%） | 103（30.2%） | 247 |  |
| Gastric Antrum | 182（40.6%） | 110（32.3%） | 292 |  |
| Anterior and posterior walls of the upper gastric body | 1（0.2%） | 2（0.6%） | 3 |  |
| Greater curvature of the lower gastric body | 0（0.0%） | 12（3.5%） | 12 |  |
| Pre-pyloric region | 29（6.5%） | 2（0.6%） | 31 |  |
| Lesion size |  |  |  | ＜0.001^*^ |
| 0 | 20（8.3%） | 0（0.0%） | 20 |  |
| <1 cm | 156（65.0%） | 39（33.6%） | 195 |  |
| ≥1 cm | 64（26.7%） | 77（66.4%） | 141 |  |
| Morphology |  |  |  | ＜0.001^*^ |
| None | 13（5.4%） | 0（0.0%） | 13 |  |
| Elevated | 36（15.0%） | 2（1.7%） | 38 |  |
| Flat | 120（50.0%） | 32（27.6%） | 152 |  |
| Depressed | 71（29.6%） | 82（70.7%） | 153 |  |
| Colour |  |  |  | 0.012^*^ |
| None | 13（5.4%） | 0（0.0%） | 13 |  |
| Same | 12（5.0%） | 2（1.7%） | 14 |  |
| Pale | 42（17.5%） | 18（15.5%） | 60 |  |
| Red | 173（72.1%） | 96（82.8%） | 269 |  |
| Erosion |  |  |  | 0.002^*^ |
| Absent | 219（91.2%） | 115（99.1%） | 334 |  |
| Present | 21（8.8%） | 1（0.9%） | 22 |  |
| Surface nodularity |  |  |  | 0.032^*^ |
| Absent | 213（88.8%） | 111（95.7%） | 324 |  |
| Present | 27（11.2%） | 5（4.3%） | 32 |  |
| Ulceration |  |  |  | 0.546 |
| Absent | 239（99.6%） | 115（99.1%） | 354 |  |
| Present | 1（0.4%） | 1（0.9%） | 2 |  |
| Clear border |  |  |  | ＜0.001^*^ |
| Absent | 188（78.3%） | 61（52.6%） | 249 |  |
| Present | 52（21.7%） | 55（47.4%） | 107 |  |
| IM patches |  |  |  | ＜0.001^*^ |
| Absent | 229（95.4%） | 88（75.9%） | 317 |  |
| Present | 11（4.6%） | 28（24.1%） | 39 |  |

Note: **P* < 0.05

Table 4 presents C-WLE lesion characteristics by OLGIM stage. Significant differences (*P* < 0.05) were found for *H. pylori* history, mucosal status, lesion number and size, morphology, border clarity, IM patches, colour, erosion, and surface nodularity. High-risk OLGIM correlated with similar C-WLE features as high-risk OLGA, reinforcing the macroscopic endoscopic markers for advanced precancerous lesions.
