# Supplementary material for: Risk prediction model for precancerous gastric lesions based on magnifying endoscopy combined with narrow-band imaging features
Source: Front Oncol. 2025 Apr 4;15:1554523. doi: 10.3389/fonc.2025.1554523 (PMC12006015; doi:10.3389/fonc.2025.1554523)
Supplement: Supplementary file 9 [file Table5.docx]

***Supplementary Material***

**Risk Prediction Model for Precancerous Gastric Lesions Based on Magnifying Endoscopy Combined with Narrow-band Imaging Features**

**Supplementary Table**

Supplementary TABLE 5. Lesion Characteristics Based on OLGIM Staging (ME-NBI findings)

| Characteristic | Low-risk OLGIM | High-risk OLGIM | Total | P-value |
| --- | --- | --- | --- | --- |
| DL |  |  |  | ＜0.001^*^ |
| Absent | 158（65.8%） | 28（24.1%） | 186 |  |
| Present | 82（34.2%） | 88（75.9%） | 170 |  |
| IMVP |  |  |  | 0.309 |
| Absent | 221（92.1%） | 103（88.8%） | 324 |  |
| Present | 19（7.9%） | 13（11.2%） | 32 |  |
| IMSP |  |  |  | 0.718 |
| Absent | 220（91.7%） | 105（90.5%） | 325 |  |
| Present | 20（8.3%） | 11（9.5%） | 31 |  |
| LBC |  |  |  | ＜0.001^*^ |
| Absent | 36（15.0%） | 1（0.9%） | 37 |  |
| Present | 204（85.0%） | 115（99.1%） | 319 |  |
| WOS |  |  |  | ＜0.001^*^ |
| Absent | 70（29.2%） | 8（6.9%） | 78 |  |
| Present | 170（70.8%） | 108（93.1%） | 278 |  |
| WGA |  |  |  | ＜0.001^*^ |
| Absent | 237（98.8%） | 105（90.5%） | 342 |  |
| Present | 3（1.2%） | 11（9.5%） | 14 |  |
| MCDL border size |  |  |  | ＜0.001^*^ |
| 0 | 157（65.4%） | 28（24.1%） | 185 |  |
| >0 to <1/3 | 4（1.7%） | 43（37.1%） | 47 |  |
| ≥1/3 to <2/3 | 8（3.3%） | 42（36.2%） | 50 |  |
| ≥2/3 | 71（29.6%） | 3（2.6%） | 74 |  |
| MCDL border regularity |  |  |  | ＜0.001^*^ |
| None | 158（65.8%） | 28（24.1%） | 186 |  |
| Regular | 75（31.2%） | 3（2.6%） | 78 |  |
| Irregular | 7（2.9%） | 85（73.3%） | 92 |  |
| VEC |  |  |  | 0.006^*^ |
| Absent | 231（96.2%） | 103（88.8%） | 334 |  |
| Present | 9（3.8%） | 13（11.2%） | 22 |  |

Note: **P* < 0.05

Table 5 details ME-NBI features and OLGIM stage. Significant differences (P < 0.001) were observed for DL, LBC, WOS, WGA, MCDL border regularity and size, and VEC pattern (P = 0.006). High-risk OLGIM exhibited increased DL, LBC, WOS, WGA, irregular MCDL border, larger MCDL size, and VEC pattern, further emphasizing ME-NBI's role in identifying microvascular and mucosal changes in advanced OLGIM stages.
